# Arsenic immobilization and greenhouse gas emission depend on quantity and frequency of nitrogen fertilization in paddy soil

**DOI:** 10.1016/j.heliyon.2024.e35706

**Published:** 2024-08-03

**Authors:** Hanna Grimm, Soeren Drabesch, Alan Nicol, Daniel Straub, Prachi Joshi, Christiane Zarfl, Britta Planer-Friedrich, E. Marie Muehe, Andreas Kappler

**Affiliations:** a Geomicrobiology, Department of Geosciences, University of Tübingen, Schnarrenbergstrasse 94-96, 72076 Tübingen, Germany; b Plant Biogeochemistry, Department of Applied Microbial Ecology, Helmholtz Centre for Environmental Research - UFZ, Permoserstrasse 15, 04318 Leipzig, Germany; c Plant Biogeochemistry, Department of Geosciences, University of Tübingen, Schnarrenbergstrasse 94-96, 72076 Tübingen, Germany; d Environmental Geochemistry, Bayreuth Center for Ecology and Environmental Research (BayCEER), University of Bayreuth, Germany; e Quantitative Biology Center (QBiC), University of Tübingen, Germany; f Environmental Systems Analysis, Department of Geosciences, University of Tübingen, Schnarrenbergstrasse 94-96, 72076 Tübingen, Germany; g Cluster of Excellence: EXC 2124: Controlling Microbes to Fight Infection, Tübingen, Germany

**Keywords:** Nitrate reduction, Iron(II) oxidation, Ferrous iron, Nitrous oxide, Methane, Global warming potential

## Abstract

Nitrogen (N) fertilization in paddy soils decreases arsenic mobility and methane emissions. However, it is unknown how quantity and frequency of N fertilization affects the interlinked redox reactions of iron(II)-driven denitrification, iron mineral (trans-)formation with subsequent arsenic (im-)mobilization, methane and nitrous oxide emissions, and how this links to microbiome composition. Thus, we incubated paddy soil from Vercelli, Italy, over 129 days and applied nitrate fertilizer at different concentrations (control: 0, low: ∼35, medium: ∼100, high: ∼200 mg N kg^−1^ soil^−1^) once at the beginning and after 49 days. In the high N treatment, nitrate reduction was coupled to oxidation of dissolved and solid-phase iron(II), while naturally occurring arsenic was retained on iron minerals due to suppression of reductive iron(III) mineral dissolution. In the low N treatment, 40 μg L^−1^ of arsenic was mobilized into solution after nitrate depletion, with 69 % being immobilized after a second nitrate application. In the non-fertilized control, concentrations of dissolved arsenic were as high as 76 μg L^−1^, driven by mobilization of 36 % of the initial mineral-bound arsenic. Generally, N fertilization led to 1.5-fold higher total GHG emissions (sum of CO_2_, CH_4_ and N_2_O as CO_2_ equivalents), 158-fold higher N_2_O, and 7.5-fold lower CH_4_ emissions compared to non-fertilization. On day 37, *Gallionellaceae*, *Comamonadaceae* and *Rhodospirillales* were more abundant in the high N treatment compared to the non-fertilized control, indicating their potential role as key players in nitrate reduction coupled to iron(II) oxidation. The findings underscore the dual effect of N fertilization, immobilizing arsenic in the short-term (low/medium N) or long-term (high N), while simultaneously increasing N_2_O and lowering CH_4_ emissions. This highlights the significance of both the quantity and frequency of N fertilizer application in paddy soils.

## Highlights

• Nitrate fertilization led to Fe(III) mineral formation and arsenic immobilization.
• Higher and repeated nitrate fertilization immobilized most arsenic.
• GHG emissions were independent of amount of nitrate but higher than without nitrate.
• Nitrate fertilization decreased CH_4_ emissions, but increased N_2_O emissions.
• Iron-oxidizing *Gallionellaceae* species enriched by nitrate fertilization.

## Graphical abstract

**Figure:**
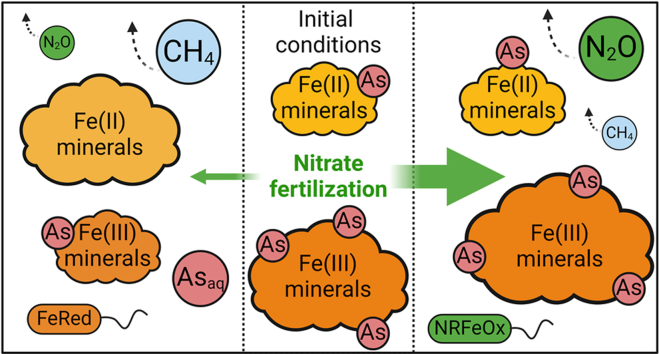

## 1 Introduction

Increases in global rice production are needed to meet the future food demand arising from a growing and developing population without simultaneously increasing harmful impacts on the climate [1]. Thus, paddy soil management strategies should balance rice yield and greenhouse gas emissions [2,3]. Paddy soils account for only 9 % of the total cropland area [4], but contribute 48 % to total cropland greenhouse gas emissions due to high methane (CH_4_) emissions (global warming potential over 100 years: 27) [5,6]. Inorganic nitrogen (N) fertilizer application was found to decrease the global warming potential by 4.2 % per unit rice yield as it mitigates CH_4_ emissions and increases rice yield [2]. Such benefits could be enhanced by using slow release fertilizer or optimized timing of N fertilizer addition [2]. However, in waterlogged, anoxic paddy soils, N fertilization stimulates denitrification accompanied by increased emissions of nitrous oxide (N_2_O) (global warming potential over 100 years: 273) [6]. Nitrate reduction is mostly limited by the amount, bioavailability, and energy yield of electron donors [[7], [8], [9]]. In addition to organic carbon as electron donor for nitrate reduction under anoxic conditions, iron(II) (Fe(II)), which is generated at high concentrations by microbial iron(III) (Fe(III)) reduction, can serve as an electron donor for nitrate-reducing, Fe(II)-oxidizing microorganisms (autotrophic denitrification) [10]. Together with the abiotic oxidation of Fe(II) by reactive N species via chemodenitrification, both processes contribute to the emission of N_2_O as well as to the formation of Fe(III) minerals [11]. Fe(III) minerals can serve as adsorption matrix for nutrients or contaminants, such as arsenic [12,13].

Arsenic is ubiquitously and naturally present in paddy soils and often found in groundwater used for irrigation. Thus, it is considered as the most important contaminant in paddy soils [14]. Its mobility and bioavailability are greatly influenced by Fe redox cycling and sequestration by Fe(III) (oxyhydr)oxide minerals [15]. Owing to waterlogged, anoxic conditions, arsenic is released during reductive Fe(III) mineral dissolution [16,17] and is reduced to its more toxic and mobile species arsenite [18]. Besides posing risks for humans by dietary uptake, accumulation of arsenic in rice plants also negatively affects plant growth and inhibits grain filling, ultimately reducing grain yield [19]. Arsenic mobility and toxicity is expected to be greatly influenced by the application of N fertilizers to paddy soils due to microbial nitrate reduction coupled to Fe(II) oxidation or chemodenitrification. These processes lead to the formation of Fe(III) minerals and provide adsorption sites for arsenic, reducing its mobility. The fate of arsenic can also be influenced by nitrate reduction coupled to arsenite oxidation, suppression of reductive dissolution of arsenic-bearing Fe(III) minerals by providing nitrate as a more favorable electron acceptor [20] or by the type of N fertilizer. Wang et al. (2023) [21] compared nitrate and ammonia-nitrate fertilizers in anoxic paddy soil microcosms and found less As immobilization for ammonia-nitrate fertilizers due to the presence of Feammox (ammonium-stimulated Fe(III) mineral reduction).

Even though the individual effects of N fertilizer addition on either arsenic mobility [20,22,23] or greenhouse gas emissions (i.e., N_2_O and CH_4_) [[24], [25], [26]] have been studied in paddy soils before, the effects of different quantities and frequencies of N fertilizer application on (1) nitrate reduction coupled to Fe(II) oxidation and on greenhouse gas formation and emission, (2) on Fe mineral formation and transformation, subsequently (3) on the mobility of arsenic, and (4) the link to microbiome composition and activity remain unresolved in paddy soils. Acknowledging the fact that mainly urea or ammonia-based fertilizers are applied to paddy fields, we supplied nitrate as a fertilizer to directly couple nitrate reduction to iron(II) oxidation by excluding confounding effects of ammonification and nitrification processes. This allows for a mechanistic understanding of the interplay between the N, Fe, As, and C cycles. In the following, N fertilization refers to fertilization with nitrate.

## 2 Materials & methods

### 2.1 Soil sampling and characterization

Paddy soil samples were collected in October 2020 in Vercelli, Italy, located in the Po river plain of Piedmont. The sampled paddy field is located on the Cascina Boraso research farm (45°19′26.0″ N 8°22′24.6″ E) at the international rice research institute (CREA-CI). It is intensively managed with LUNA-CL, a Long A grain rice cultivar (Clearfield®) under waterlogged conditions. Fertilizers are applied in excess in the region of Piedmont [27]. In 2020, a total of 279 kg N ha^−1^ (∼105 mg N kg^−1^ soil^−1^) were applied as urea at pre-sowing (24 %), as 1st (24 %) and 2nd (52 %) top dressing. Paddy soil samples were taken with a shovel after removal of the plant layer from the upper 20 cm and stored at 4 °C in the dark until further processing. Soil characterization comprised analyses of soil texture, bulk density, water content, pH, cation exchange capacity, total elemental content, total organic carbon and total N content, water-extractable organic carbon and inorganic N species and sequentially extractable Fe and arsenic (1 M sodium acetate, 0.5 M HCl, 6 M HCl) (Supporting methods S1, Table SI 1).

### 2.2 Incubation experiment

#### 2.2.1 Setup and pre-incubation

For microcosm experiments, serum bottles (245 mL total volume) were washed with 1 M HCl (10 min), rinsed three times with deionized water and sterilized at 180 °C for 4.5 h. Fresh paddy soil (25 ± 0.05 g) was weighed into each serum bottle (in total 15 bottles) under sterile conditions and degassed with N_2_ (for our experiments, no arsenic was added; only the naturally occurring arsenic content was considered). Sub-samples were taken in triplicates during filling of serum bottles and dried at 70 °C for 72 h to determine the soil moisture content. Artificial irrigation water (200 mL, Table SI 2) was added under N_2_ atmosphere to each serum bottle and microcosms were pre-incubated without any addition of N fertilizer for 18 days at 25 °C in the dark to acclimate the paddy soil, to deplete soil-borne nitrate, and to build up dissolved Fe(II) (Fig. SI 1). To prevent accumulation of produced gases in the microcosms, the headspace was continuously flushed through a sterile 0.22 μm filter (polyethersulfone membrane, Carl Roth GmbH + Co. KG, Germany) with pre-moistened N_2_ gas to minimize evaporation and water loss (Fig. SI 1). Three serum bottles were sampled during the course of the pre-incubation, while the others were not disturbed.

#### 2.2.2 Fertilization and re-fertilization

After 18 days of pre-incubation, microcosms were subjected to different N fertilization regimes in triplicates (control: no N addition, low: ∼35 mg N kg^−1^ soil^−1^, medium: ∼100 mg N kg^−1^ soil^−1^, high: ∼200 mg N kg^−1^ soil^−1^) by the addition of potassium nitrate (KNO_3_, quality level: MQ300, Merck KGaA, Germany) and incubated under the same conditions as described earlier (Table SI 3). After 49 days of incubation, all microcosms were re-fertilized with KNO_3_. Levels and timing of N fertilization reflect common practices in paddy soil management simulating single events of N fertilizer addition and total N fertilizer concentrations [28].

#### 2.2.3 Sampling

The microcosms were sampled for greenhouse gas emissions (CO_2_, N_2_O, CH_4_), aqueous geochemistry (N species, Fe species, arsenic species, dissolved organic carbon (DOC), pH), mineralogy (sequential chemical extractions), and microbial community analyses (Fig. SI 2). For gas sampling, the gas flow was stopped and 2–5 mL of headspace was collected in the beginning (t0) and after a 0.5–3 h period (t1). This was repeated two times. The gas samples were injected into helium-flushed exetainer® vials (12 mL, Labco Limited, United Kingdom). Sample volume and incubation time was adjusted within the course of the experiment to account for changes in headspace volume. For geochemical analyses, 2 mL of soil slurry were sampled under N_2_ atmosphere and centrifuged (5 min, 13,400 rpm). The supernatant was diluted in anoxic 1 M HCl/40 mM sulfamic acid (to prevent oxidation of Fe(II) by nitrite) [29] for Fe and arsenic species analysis and in anoxic MQ water for N species analysis. The soil pellet was dried under anoxic conditions and used for sequential chemical extractions as described below. At several timepoints, samples were taken for pH, DOC, and microbial community analyses.

#### 2.2.4 Sequential extraction

To quantify different Fe mineral phases and the associated arsenic, dried soil pellets were extracted using 0.5 M HCl, targeting poorly crystalline Fe(III) (oxyhydr)oxides and 6 M HCl to determine crystalline Fe minerals [[30], [31], [32]]. In contrast to the general soil characterization, a sodium acetate extraction was not performed, however, adsorbed Fe, Fe in amorphous sulfides and carbonates (as targeted by sodium acetate extraction), and associated arsenic were also extracted by 0.5 M HCl. Therefore, the 0.5 M HCl extraction used in the experiment is comparable to the sum of sodium acetate extractable and 0.5 M HCl extractable Fe and arsenic of the soil characterization. First, 2 mL of 0.5 M HCl/40 mM sulfamic acid were added to the dried soil pellet, the sample was well mixed and extracted under anoxic conditions for 2 h in the dark at room temperature. Sulfamic acid was added to eliminate the abiotic reaction of nitrite with Fe(II) during acidification [29]. Afterwards, the sample was centrifuged (5 min, 13,400 rpm), the supernatant diluted in 1 M HCl, and transferred and stored anoxically in the dark at 5 °C. Subsequently, 2 mL of 6 M HCl was added to the soil pellet and extracted anoxically for 24 h in the dark at room temperature. Finally, the sample was centrifuged (5 min, 13,400 rpm), the supernatant diluted in 1 M HCl, transferred and stored anoxically in the dark at 5 °C.

### 2.3 Geochemical analyses

Nitrate (NO_3_^−^), nitrite (NO_2_^−^), and ammonium (NH_4_^+^) were analyzed in the supernatant of the sampled and centrifuged soil slurry by a segmented flow analyzer (AutoAnalyzer3, SEAL Analytical, Germany), equipped with a dialysis membrane for Fe removal to prevent side reactions during analysis.

Total Fe and Fe(II) were determined in the supernatant of the sampled soil slurry and after sequential extractions using the ferrozine assay [33], following a revised protocol for nitrite-containing samples [29]. Samples were analyzed in triplicates at 562 nm on a spectrophotometer (Thermo Scientific™ Multiskan™ Go Microplate Spectrophotometer).

Samples for total arsenic after sequential extractions were analyzed by ICP-MS (Agilent 7900, USA) in argon with a helium flow of 1–3 mL min^−1^ (Table SI 4). The Agilent internal standard mix (product #5188–6525, 100 ± 5 %) and the Agilent Environmental Calibration Set (product #5183-4688) were used. Arsenic speciation in the supernatant was analyzed by ICP-MS/MS (Agilent 8900) as AsO^+^ (*m/z* 91) using oxygen as reaction cell gas. Arsenic species were separated in a high-pressure liquid chromatograph (Agilent 1260 Infinity II) equipped with a PRP-X100 column (Hamilton, 20 mM NH_4_H_2_PO_4_, flow rate 1 mL min^−1^). Quantification was done via calibration with commercial standards for arsenite and arsenate (Honeywell Fluka™, USA) and quality was verified by recovery of certified reference material (TMDA 54.6, Environment Canada 100 ± 6 %). Arsenite and arsenate concentrations made up 94.8 ± 23.9 % of the total arsenic concentrations, methylated arsenic species (monomethylarsonic acid and dimethylarsinic acid) were not detected and thiolated arsenic species could not be analyzed due to sample acidification (Table SI 5).

The pH was measured in the soil slurry using a benchtop pH meter (SG2, Mettler-Toledo GmbH, Germany) equipped with a pH electrode (InLab Easy DIN, Mettler-Toledo GmbH, Germany). DOC from the sampled, centrifuged soil slurry was analyzed by combustion at 750 °C (Elemental analyzer, multi N/C 2100S, Analytik Jena GmbH, Germany).

### 2.4 Greenhouse gas analysis

Gas samples were analyzed on a TraceGC1300 (ThermoFisher Scientific, modified by S + HA analytics), in which the sample is split into two different column configurations each connected to a pulsed discharge detector (first configuration: 30 m long, 0.53 mm ID TGBondQ column and 30 m long, 0.53 mm ID Molsieve column; second configuration: 30 m long, 0.53 mm ID TGBondQ column and a 30 m long 0.25 mm ID TGBondQ+ column (all ThermoFisher Scientific)) (Table SI 6). Gas emission rates were determined by performing linear regression analysis between gas concentrations and incubation times at three specific time points. The initial time point, t0, was calculated as the average of two initial gas measurements. The second time point, t1, corresponded to the end of the first incubation period. The third time point, t2, was derived by summing the end point measurements of both incubation periods. Cumulative emissions were then calculated from individual gas fluxes and the time intervals between measurements [34].

### 2.5 DNA- and RNA-based microbial community analysis

Soil samples were frozen in liquid N_2_ and stored at −80 °C prior to extraction. Total RNA and DNA were co-extracted from soil samples using a phenol-chloroform extraction protocol [35]. Quality and quantity of extracted DNA and RNA were determined using NanoDrop (NanoDrop 1000, Thermo Scientific, Waltham, MA, USA), gel electrophoresis (8 out of 78 samples randomly selected), and Qubit (Life Technologies, Carlsbad, CA, USA), respectively. DNA was digested using the TURBO DNA-free™ Kit to obtain pure RNA samples with subsequent reverse transcription using SuperScript™ III Reverse Transcriptase to obtain complementary DNA (cDNA). Quantitative PCR for DNA and cDNA was performed for bacterial 16S rRNA genes and different marker and functional genes using SybrGreen® Supermix (5 μL per qPCR reaction, Bio-Rad Laboratories GmbH, Munich, Germany) in addition to dimethylsulfoxide (DMSO, 0.5 μL per qPCR reaction, Carl Roth) on the C1000 Touch thermal cycler (CFX96TM real time system). For 16S rRNA gene amplicon sequencing, the 16S rRNA gene was amplified using primers 515f (GTGYCAGCMGCCGCGGTAA) [36] and 806r (GGACTACNVGGGTWTCTAAT) [37] targeting the V4 region. Sequencing data was analyzed using the nf-core/ampliseq pipeline (v2.3.1), which encompasses all necessary analysis steps and software. The pipeline is publicly available [38,39], and was executed with Nextflow (v21.10.3) [40] and Singularity (v3.8.7) [41]. Details of quantitative PCR analysis and Illumina sequencing can be found in the Supporting methods S2 and Table SI 7.

### 2.6 Data analysis

A non-parametric Kruskal-Wallis test was applied using R (4.3.3) and its interface RStudio (2023.12.1 + 402) to estimate differences in total GWP, CO_2_, CH_4_ and N_2_O emissions between treatments. A one-way analysis of variance (ANOVA) combined with a post-hoc test (Tukey test) was applied to identify differences in microbial community composition between soil treatments.

## 3 Results and discussion

### 3.1 Nitrogen fertilization stimulates nitrate reduction coupled to iron(II) oxidation

**Fig. 1:**
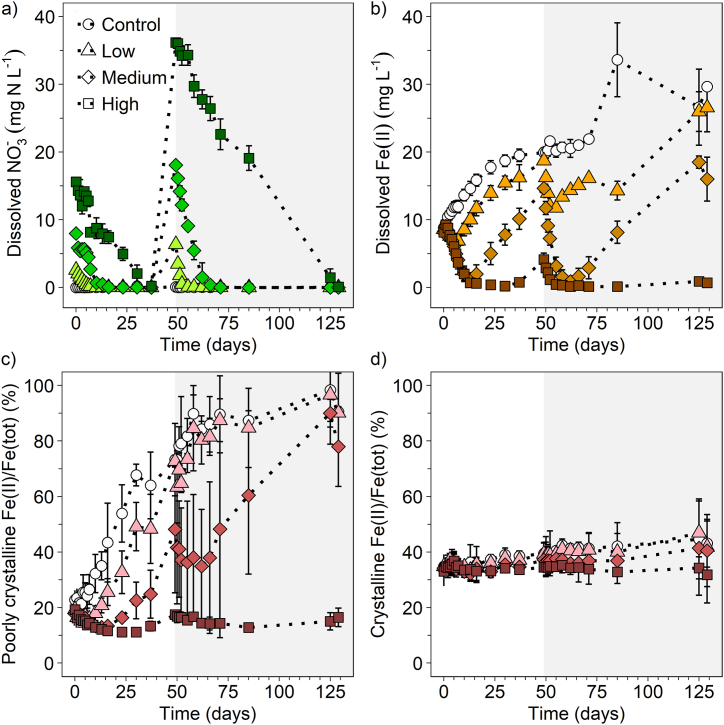
Dissolved nitrate concentrations in mg N L^−1^ (a), dissolved Fe(II) concentrations in mg L^−1^ (b), Fe(II)/Fe(tot) ratio in % in poorly crystalline Fe mineral phases (0.5 M HCl extraction) (c) and Fe(II)/Fe(tot) ratio in % in crystalline Fe mineral phases (6 M HCl extraction) (d) for three different levels of nitrogen fertilizer applications and a non-fertilized control (control: white circles, low N: triangles, medium N: diamonds, high N: squares) over 129 days of incubation. Mean ± standard deviation is shown for biological triplicates and the mean ± range for the low N treatment in the second fertilization for biological duplicates. The white background illustrates the first (0–49 days) and the grey background the second (49–129 days) nitrate fertilization period. (For interpretation of the references to colour in this figure legend, the reader is referred to the Web version of this article.)

After pre-incubation of the paddy soil microcosms for 18 days, all initial soil-borne nitrate was depleted and dissolved Fe(II) was generated (Fig. SI 3 a, b). After applying nitrate at low, medium, and high N concentrations at the beginning of the incubation, 2.6 ± 0.1, 8.0 ± 0.2 and 15.6 ± 0.1 mg N L^−1^ were completely consumed (≤0.2 mg N L^−1^) within 10, 16 and 37 days, respectively (Fig. 1a). In the second fertilization period (49–129 days), dissolved nitrate concentrations were generally higher compared to the first fertilization period (0–49 days) and declined within 6, 22 and 80 days (after the second fertilization at day 49) to below 0.1 mg N L^−1^ in the low, medium and high N treatment, respectively (Fig. 1a). In the non-fertilized control, dissolved nitrate concentrations stayed constantly below 0.2 ± 0.2 mg L^−1^ over the 129 days of incubation (Fig. 1a). We applied a pseudo-first-order kinetic model to the N fertilized treatments (Supporting methods S3, Table SI 8) and observed that at least the low and medium N treatment adhere to this model. The half-lives for nitrate reduction were higher in the medium N (0–16 days: t_1/2_ = 2.98 days, 49–71 days: t_1/2_ = 2.91 days) compared to the low N treatment (0–10 days: t_1/2_ = 1.73 days, 49–55 days: t_1/2_ = 0.92 days). A lower half-life time in the second fertilization period observed in the low N treatment likely indicates differences in for example Fe(II) concentrations and availability or microbial composition and activity compared to the first fertilization period. However, nitrate reduction in the high N treatment did not visually follow a pseudo-first-order kinetic model, indicating that nitrate is not the sole rate-determining factor at higher N application rates. This suggests that other factors, such as Fe(II) concentrations, organic carbon availability, or specific microbial activities, significantly influence the nitrate reduction process. These factors may interact in complex ways, leading to non-linear kinetics that a simple first-order model cannot capture.

Dissolved Fe(II) concentrations decreased simultaneously with nitrate concentrations. Within the first fertilization period, the greatest decrease in dissolved Fe(II) concentrations was observed in the medium N treatment (6.9 ± 0.8 mg L^−1^, between days 0 and 13) and in the high N treatment (7.9 ± 0.8 mg L^−1^, between days 0 and 30) and to a smaller extent in the low N treatment (1.8 ± 0.3 mg L^−1^, 0–6 days) (Fig. 1b). In the second fertilization period, the decrease in dissolved Fe(II) was higher in the low and medium N treatment, but lower for the high N treatment compared to the first fertilization period, due to generally low concentrations of dissolved Fe(II) in the high N treatment (Fig. 1b). In the non-fertilized control, dissolved Fe(II) concentrations increased to a maximum of 33.6 ± 5.5 mg L^−1^ on day 85 and stayed relatively constant until the end of incubation (Fig. 1b). Generally, dissolved Fe(II) concentrations increased in all fertilized treatments as soon as nitrate was depleted, indicating microbial Fe(III) reduction. In the first fertilization period, dissolved Fe(II) concentrations increased to a similar extent in the low (12.1 ± 0 mg L^−1^, 6–49 days) and medium (13.4 ± 0.4 mg L^−1^, 13–49 days) N treatment and to a lower extent in the high N treatment (3.9 ± 1 mg L^−1^, 30–49 days) (Fig. 1b). In the second fertilization period, the increase in dissolved Fe(II) concentrations after nitrate consumption was similar for the low and medium N treatment, but only minor for the high N treatment (0.8 ± 0.1 mg L^−1^, 85–125 days).

To capture Fe mineral dynamics, sequential extractions of the paddy soil were performed. In the poorly crystalline Fe mineral fraction, the Fe(II)/Fe(tot) ratio increased from 8.7 ± 1.8 % to 28.4 ± 4.5 % within 18 days of pre-incubation (Fig. SI 4). In the first fertilization period, the Fe(II)/Fe(tot) ratios generally decreased in the poorly crystalline Fe mineral fraction for all fertilized treatments, to the greatest extent for the high N treatment from 19.2 ± 3.2 to 11.2 ± 0.6 % (Fig. 1c). Increasing Fe(II)/Fe(tot) ratios were observed after nitrate depletion, resulting in Fe(II)/Fe(tot) ratios of 72.7 ± 2.1 % (low N), 48.2 ± 22.9 % (medium N) and 17.5 ± 1.5 % (high N) at the end of the first fertilization period (49 days). In the second fertilization period, Fe(II)/Fe(tot) ratios first decreased, followed by an increase again after nitrate consumption leading to high Fe(II)/Fe(tot) ratios in the low (90.1 ± 1.4 %) and medium N treatment (78 ± 14.4 %) at the end of incubation (129 days). Increasing Fe(II)/Fe(tot) ratios point towards new, highly reactive, and bioavailable Fe(II) minerals formed by Fe(III) reduction. For the high N treatment, Fe(II)/Fe(tot) ratios stayed relatively constant at 13–17 % throughout the second fertilization period, which is likely caused by the lack of bioavailable Fe(II) minerals, as they had already been oxidized during the first phase of fertilization and were not recycled by Fe(III) reduction as in the other fertilized treatments. Fe(II)/Fe(tot) ratios in the poorly crystalline Fe mineral fraction of the non-fertilized control increased continuously from 22.8 ± 3.4 % to 90.5 ± 13.9 % over the 129 days of incubation (Fig. 1c). In the crystalline Fe mineral fraction, Fe(II)/Fe(tot) ratios stayed constant at ∼35 % over 18 days of pre-incubation (Fig. SI 4). Within the first and second fertilization period, Fe(II)/Fe(tot) ratios in the crystalline mineral fraction were less prone to large changes compared to the poorly crystalline Fe mineral fraction (Fig. 1d). Yet, the ratio slowly increased constantly for the non-fertilized control (34.8 ± 4.3 % to 43.2 ± 8.6 %) as well as for the low (34.9 ± 3.6 % to 41.9 ± 9.3) and medium (34.2 ± 1.5 % to 40.5 ± 13 %) N treatment within 129 days of incubation, generating more crystalline Fe(II) minerals in the long-term. The constant level of Fe(II)/Fe(tot) ratios in the crystalline Fe mineral fraction might be attributed to less bioavailable Fe minerals, such as magnetite, Fe(II)-bearing silicates or sulfides [32], that are stable even under redox fluctuations [42,43]. In contrast to the medium and low N treatment, the Fe(II)/Fe(tot) ratio remained relatively stable for the high N treatment at 32–33 %.

Overall, nitrate fertilization led to a microbial coupling of nitrate reduction to Fe(II) oxidation [20,[44], [45], [46], [47]], evidenced by the simultaneous decrease of nitrate and dissolved Fe(II) concentrations, as well as the decrease of the Fe(II)/Fe(tot) ratios in the poorly crystalline Fe mineral fraction after applying nitrate fertilizer. Based on the concentrations of dissolved nitrate and Fe(II), we calculated that the oxidation of dissolved Fe(II) was responsible for a maximum of 5.6 % of reduced nitrate (Table SI 9). The remainder was most likely caused by solid-phase Fe(II) pools, accounting for the larger proportion of Fe(II). Due to soil heterogeneity and the resulting variations in the total Fe and Fe(II) concentrations, calculations based on absolute values of oxidized solid-phase Fe(II) are not applicable. The decrease in Fe(II)/Fe(tot) ratio was likely caused by the oxidation of dissolved Fe(II), leading to the formation of Fe(III) minerals, and by the oxidation of solid-phase Fe(II) minerals coupled to nitrate reduction. The oxidation of solid-phase Fe(II) minerals (e.g., siderite, pyrite, green rust, reduced goethite, biotite) by nitrate-reducing, Fe(II)-oxidizers was also observed in other studies before [[48], [49], [50], [51]]. Fe(II) was likely the major electron donor responsible for nitrate reduction as no other potential electron donor (e.g., organic carbon [52], ammonium [53], CH_4_ [54], etc.) showed a clear relationship with nitrate concentrations (Fig. SI 5, 6, 7). Even though heterotrophic and autotrophic Fe(II)-driven denitrification can co-occur in paddy soils, we suggest that autotrophic Fe(II)-driven denitrification is the dominant process in our study, which is supported by decreasing dissolved Fe(II) concentrations, 0.5 M HCl extractable Fe(II)/Fe(tot) ratio, nitrate concentrations, and the lack of bioavailable fatty acids (representative samples analyzed by HPLC). Our data shows that as long as nitrate was present in solution, microbial Fe(III) mineral reduction was inhibited as nitrate is the thermodynamically more favorable electron acceptor relative to Fe(III) minerals [11,55,56]. Only after nitrate was completely consumed, Fe(III) reduction dominated and resulted in increasing dissolved Fe(II) concentrations and increasing Fe(II)/Fe(tot) ratios in the poorly crystalline Fe mineral fraction. Poorly crystalline Fe minerals were identified to be highly susceptible to redox changes induced by the application of N fertilizer, highlighting that crystallinity and bioavailability of Fe minerals impact the extent of Fe(II) oxidation.

### 3.2 Arsenic immobilization by iron minerals formed by nitrogen fertilization

**Fig. 2:**
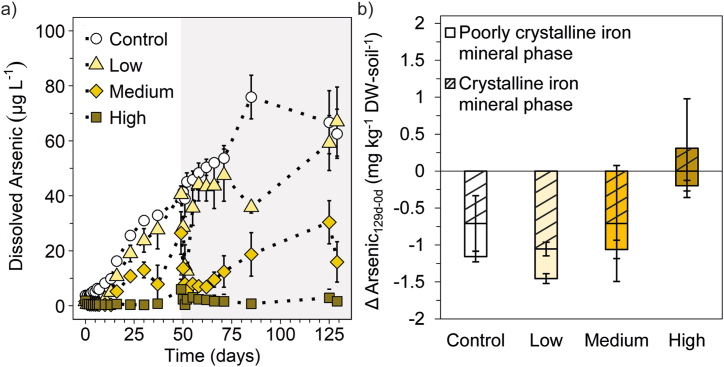
Dissolved arsenic concentrations in μg L^−1^ (a), and difference in arsenic content associated with Fe mineral phases between day 0 and 129 in mg kg^−1^ DW-soil^−1^ (b) in paddy soil with three different levels of nitrogen fertilizer applications (low, medium, high) compared to the non-fertilized control over 129 days of incubation. Mean ± standard deviation is shown for biological triplicates and the mean ± range for the low N treatment in the second fertilization for biological duplicates. The white background in (a) illustrates the first (0–49 days), and the grey background the second (49–129 days) nitrate fertilization period.

We analyzed naturally occurring arsenic in the paddy soil to evaluate changes in arsenic mobility over the course of incubation. Total dissolved arsenic concentrations (calculated as the sum of arsenite and arsenate) stayed constantly low in the high N treatment between 0.4 ± 0.1 and 6 ± 3.7 μg L^−1^ over the 129 days of incubation (Fig. 2a). In the low and medium N treatment, total dissolved arsenic concentrations started to increase in both fertilization periods after nitrate was completely consumed. Dissolved arsenic concentrations reached 40.6 ± 3.1 μg L^−1^ (low N) and 26.5 ± 7.7 μg L^−1^ (medium N) at the end of the first fertilization period (after 49 days) and decreased by 27.8 ± 3.3 μg L^−1^ (low N) and by 21.1 ± 7.7 μg L^−1^ (medium N) within 3 days after the second N fertilizer application. At the end of incubation after 129 days, dissolved arsenic concentrations were lower in the medium N treatment (16 ± 7.4 μg L^−1^) compared to the low N treatment (67 ± 12.5 μg L^−1^). Total dissolved arsenic concentrations increased continuously in the non-fertilized control from 3.8 ± 0.5 μg L^−1^ to 62.6 ± 8.9 μg L^−1^ over the 129 days of incubation.

Arsenite initially constituted approximately 15–30 % of the total dissolved arsenic (70–85 % arsenate) and was removed completely from solution within 1 or 2 days after firstly applying N fertilizer (Fig. SI 8 a-d, Fig. SI 9). Even though absolute arsenate concentrations also decreased, arsenate accounted for 100 % of the remaining dissolved arsenic. After 10, 16 and 37 days, dissolved arsenite and arsenate concentrations started to increase in the low, medium and high N treatment, respectively, which correlated with the time of nitrate depletion. At the end of incubation (129 days), arsenite made up 48–67 % of total dissolved arsenic in the N fertilized treatments (33–52 % arsenate), with the highest proportion of arsenite observed in the high N treatment. After the second fertilizer application, the proportion of arsenite did not decline to the same extent as observed 1 or 2 days after the first fertilizer application. The non-fertilized control showed a gradual increase of dissolved arsenite and arsenate, with arsenite accounting for 30–40 % within the 129 days of incubation.

To investigate the role of Fe mineral phases for the mobility of arsenic, total arsenic concentrations associated with the poorly crystalline and crystalline Fe mineral phases were quantified (Fig. 2b). Over the 129 days of incubation, arsenic was lost for all treatments from the poorly crystalline Fe mineral phases in comparison to initial mineral-bound arsenic contents. The decline in bound arsenic was the greatest for the non-fertilized control (0.5 ± 0.1 mg kg^−1^ DW-soil^−1^), low (0.4 ± 0.1 mg kg^−1^ DW-soil^−1^) and medium N treatment (0.4 ± 0.1 mg kg^−1^ DW-soil^−1^). The least arsenic was lost from poorly crystalline Fe minerals in the high N treatment (0.2 ± 0.1 mg kg^−1^ DW-soil^−1^). The loss of arsenic from the crystalline Fe mineral fraction was about 2-fold greater for the non-fertilized control (0.7 ± 0.4 mg kg^−1^ DW-soil^−1^), low (1.1 ± 0.1 mg kg^−1^ DW-soil^−1^) and medium (0.7 ± 0.8 mg kg^−1^ DW-soil^−1^) N treatment in comparison to the loss from the poorly crystalline phase. The high N treatment even exhibited an increase of 0.3 ± 0.7 mg kg^−1^ DW-soil^−1^ in arsenic content in the crystalline Fe mineral phase.

These results illustrate that arsenic mobility is tightly linked to the fate of Fe minerals, and thus, susceptible to Fe redox changes [57]. When nitrate reduction coupled to Fe(II) oxidation dominates after N fertilizer application, dissolved arsenic can be scavenged within only a few days and retained on Fe mineral phases, even though only for a short time under low and medium N fertilizer applications. Liu et al. (2022) [20] observed that the retention of arsenic by Fe minerals is only of short-term duration (1–3 days) when applying KNO_3_ as fertilizer. In contrast, the high N treatment successfully retained arsenic on Fe mineral phases limiting mobilization also over long term (129 days). When no nitrogen fertilizer was applied, 36 % of the total arsenic was mobilized from Fe mineral phases to the solution, which co-occurred with Fe(III) reduction. Even though the poorly crystalline Fe mineral phase was influenced to a greater extent by nitrogen fertilization, more arsenic was released from the crystalline Fe mineral phase. This might be due to the larger pool of Fe minerals in the crystalline fraction compared to the poorly crystalline fraction and generally higher amounts of arsenic being asssociated with the crystalline Fe mineral phase (Table SI 1).

Other mechanisms affecting arsenic mobility include nitrate reduction coupled to arsenite oxidation, which could be occurring under N fertilization [58]. This likely caused the strong decrease of the arsenite share on the total dissolved arsenic pool after N fertilization, pointing towards a greater removal of arsenite, especially in the first fertilization period. Dissolved arsenate concentrations decreased due to the preferential adsorption of arsenate to the newly formed Fe(III) (oxyhydr)oxides under given pH's (Fig. SI 10) [[13], [59]]. In the second fertilization phase, a less pronounced oxidation effect of arsenite may be associated with generally elevated concentrations of both arsenite and arsenate, or it could be influenced by potential toxicity effects, thereby impeding microbial processes. As the binding of As to Fe(III) (oxyhydr)oxides is dependent on various parameters, such as mineral identity, properties, and structure, or pH, additional analysis would be required to elucidate the binding environment of As and Fe more in detail [59,60].

In summary, N fertilization successfully removed arsenite from solution and immobilized arsenic by adsorption onto newly formed Fe(III) minerals, even though the extent and efficiency depends on the amount of N fertilizer added. Reductive dissolution of Fe(III) minerals was responsible for arsenic mobilization, especially of arsenite, mainly from the crystalline mineral phase. However, redox cycling or type of binding of arsenic species also likely play a role for the mobilization of arsenic [61].

### 3.3 Greenhouse gas emissions depend on nitrogen fertilizer concentrations

**Fig. 3:**
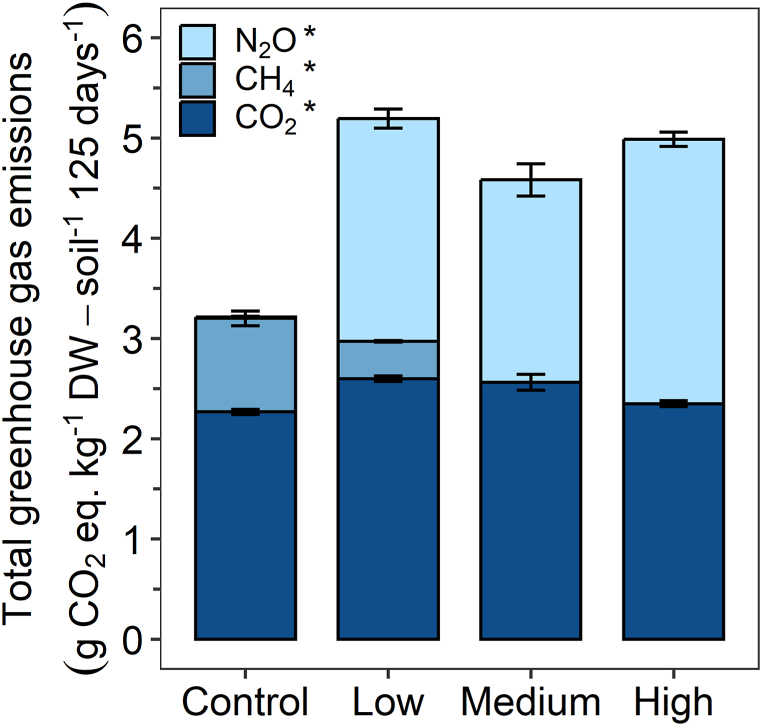
Total greenhouse gas emissions in g CO_2_ eq. kg^−1^ DW-soil^−1^ 125 days^−1^ from CO_2_ (dark blue), CH_4_ (blue), N_2_O (light blue) emissions for the three different levels of nitrogen fertilizer application (low, medium, and high N) compared to the non-fertilized control. CO_2_ equivalents of CH_4_ and N_2_O emissions were calculated by multiplication with the factors 27 and 273, respectively, which represent the global warming potential over 100 years [6]. Asterisks (*) represent significant differences (p < 0.05) of total CO_2_, CH_4_ and N_2_O emissions between treatments. Mean ± standard deviation is shown for biological triplicates taking the mean ± range for the low N treatment in the second fertilization for biological duplicates into account. (For interpretation of the references to colour in this figure legend, the reader is referred to the Web version of this article.)

To evaluate climate-related effects of N fertilizer application, CO_2_, CH_4_, and N_2_O emissions were quantified over the course of incubation as CO_2_ equivalents [6]. Total greenhouse gas emissions were similar for the low, medium and high N treatment (5.2 ± 0.2, 4.6 ± 0.4, 5.0 ± 0.1 g CO_2_ eq. kg^−1^ DW-soil^−1^ 125 days^−1^, respectively) and higher than the non-fertilized control (3.2 ± 0.1 g CO_2_ eq. kg^−1^ DW-soil^−1^ 125 days^−1^) (Fig. 3), although not significantly (p = 0.06, non-parametric Kruskal-Wallis test, Table SI 10).

The contributions of the individual greenhouse gases to the total emissions varied between the different treatments. CO_2_ emissions were significantly different between the treatments (p = 0.04, non-parametric Kruskal-Wallis test, Table SI 10) and accounted for the largest proportion of total greenhouse gas emissions in the non-fertilized control (70.5 ± 3.4 %) compared to the N fertilized treatments (low: 50.1 ± 2.5 %, medium: 56 ± 6.3 %, high: 47.1 ± 1.6 %) (Fig. SI 11). CH_4_ emissions were significantly different between treatments (p = 0.02, non-parametric Kruskal-Wallis test, Table SI 10) and only observed for the non-fertilized control and low N treatment, contributing 29 ± 4.7 % and 7.2 ± 0.5 % to the total greenhouse gas emissions, respectively (Fig. SI 12). CH_4_ emissions were absent in the medium and high N treatment, even after nitrate was depleted. N_2_O emissions were also significantly different between treatments (p = 0.03, non-parametric Kruskal-Wallis test, Table SI 10) and mainly observed for the fertilized treatments as long as nitrate was present in the soil (Fig. SI 13), without great differences between the amount of nitrate fertilizer added. In total, N_2_O made up for 42.8 ± 4.1 % (low N), 44.0 ± 8.1 % (medium N) and 52.9 ± 3.1 % (high N) of the total greenhouse gas emissions. In contrast, only 0.5 ± 0.5 % were emitted as N_2_O in the non-fertilized control (Fig. SI 13). However, it has to be noted that only gaseous N_2_O concentrations are considered and might be underestimated as we left microcosms undisturbed before gas sampling.

N_2_O and CH_4_ emissions showed an inverse relationship, supporting prior studies that N fertilization suppresses methanogenesis [62,63]. In the control treatment, CH_4_ emissions occurred after around 50 days of incubation. The timing likely represents conditions in the soil favoring methanogenesis, meaning that the soil was depleted in other, more favorable electron acceptors, i.e. nitrate, Fe(III), and sulfate. Due to the absence of nitrate, N_2_O emissions were generally low. In contrast, N_2_O was produced when nitrate was applied to the paddy soil, due to nitrate reduction coupled to Fe(II) or As(III) oxidation and other labile organic carbon sources or abiotic processes, i.e. chemodenitrification. CH_4_ emissions were suppressed under N fertilization likely due to thermodynamic constraints (nitrate reduction being more favorable over methanogenesis), yet, nitrate reduction coupled to methane oxidation could have also limited CH_4_ emissions [64]. It was estimated that methane oxidation coupled to nitrate reduction could offset 10–20 % of the global CH_4_ emissions [65]. Vaksmaa et al. (2016) [66] showed that this process is contributing substantially to methane oxidation in an Italian paddy soil, from where our paddy soil also originates from. Together with relative abundances (based on 16S rRNA gene amplicon sequencing) of *Candidatus Methanoperedens* that are significantly higher (Welch *t*-test, p = 0.005, df = 2.93, t = −7.63, 95 % confidence interval = −0.31;-0.13) in the high N treatment (0.57 ± 0.04 %) compared to the control (0.35 ± 0.02 %) on day 37, methane oxidation coupled to nitrate reduction might have also limited CH_4_ emissions in the N fertilized treatments in our study.

In general, N fertilization led to 1.5-fold greater total greenhouse gas emissions compared to the non-fertilized control, mainly due to overall greater N_2_O emission. However, the extent of emissions was independent of the concentration of N fertilizer added, which might be due to the limited supply or bioavailability of electron donors [67] or in general the abundance and metabolic activity of the microorganisms present. When comparing different levels of N fertilization, we conclude that lower application quantities, but higher frequencies could even enhance total greenhouse gas emission (e.g., low compared to high N treatment), especially due to short-term peak emissions of N_2_O [68].

### 3.4 Change in microbial community composition by nitrogen fertilization

**Fig. 4:**
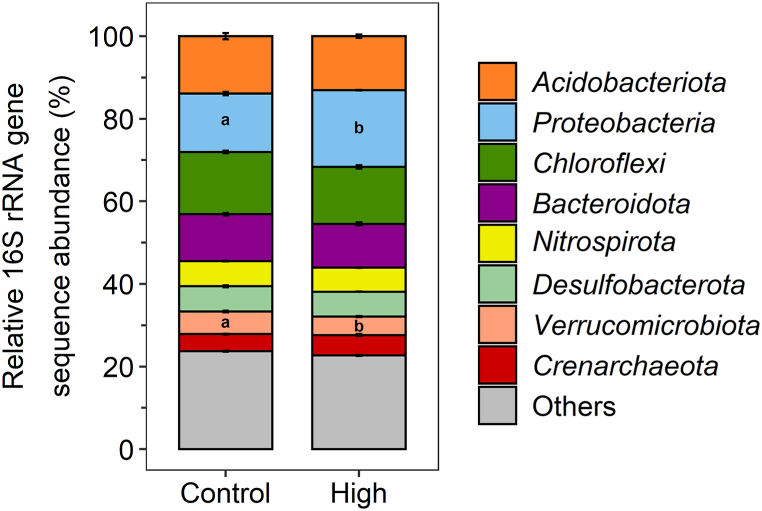
Relative 16S rRNA gene sequence abundance in % on phylum level for the non-fertilized control and high N treatment on day 37. “Others” represent phyla with abundances below 3 % on average. Significant differences between non-fertilized control and high N treatment are indicated with a and b (*Verrucomicrobiota*: p = 0.044, *Proteobacteria*: p = 1.5·10^−6^, one-way ANOVA). Mean ± standard deviation is shown for biological triplicates.

In order to identify impacts of N fertilization on the microbiome, we quantified microbial community abundance, activity and composition. The 16S rRNA gene and transcript copy numbers generally varied between different timepoints during the incubation (Fig. SI 14, 15). The greatest differences between treatments were found on day 37 between the non-fertilized control and the high N treatment, when the greatest differences in geochemistry (i.e., dissolved Fe(II) and arsenic, solid-phase Fe) could also be observed. 16S rRNA transcript copy numbers were significantly lower for the non-fertilized control compared to the high N treatment on day 37 (p = 0.0167, Table SI 11, ANOVA). Abundances of functional and marker genes seemed to be similar to the general trends of 16S rRNA gene copy numbers. No significant differences in the abundances were found between the different N treatments on day 0, 16, 37, 49 or 129 for the marker and functional genes *narG*, *nosZ*, *aioA*, *arrA* and *Geobacter* spp. (Fig. SI 16 to 21). However, copies of genes involved in Fe or N cycling were generally higher for the medium and high N treatment compared to the low N treatment and the control on day 37, especially for *narG*, *nosZ* and *Geobacter* spp. Feng et al. (2023) [69] reported a higher abundance of the arsenite oxidase gene *aioA* in N fertilized paddy soils, yet, the similarity in *aioA* abundances between the non-fertilized control and N fertilized treatments suggests a minor role of microbial arsenite oxidation in our study. Stable trends in functional and marker genes suggest that the microbial community is resilient to nitrate fertilization, likely due to its composition being established over years of nitrogen fertilization. However, 16S rRNA gene amplicon sequencing on day 37 revealed differences in the microbial community composition on the phylum level between the non-fertilized control and the high N treatment (Fig. 4). The relative 16S rRNA gene sequence abundance of *Verrucomicrobiota* was significantly higher for the non-fertilized control (5.5 ± 0.5 %) compared to the high N treatment (4.5 ± 0.3 %) (p = 0.044, Table SI 11, one-way ANOVA), whereas *Proteobacteria* were present in greater relative abundance in the high N treatment (18.6 ± 0.1 %) compared to the non-fertilized control (14.1 ± 0.8 %) (p = 1.5·10^−6^, Table SI 11, one-way ANOVA). At the family level, *Pedosphaeraceae* belonging to *Verrucomicrobiota* were enriched in the non-fertilized control and have been found to be involved in the CH_4_-, N- and Fe-cycle (Fig. SI 22) [70]. They were also found in arsenic-contaminated soils, potentially involved in toxic metal resistance [71]. Moreover, *Pedosphaeraceae* likely play a role in methane oxidation, which could be coupled to Fe(III) reduction [72], impacting arsenic mobility and greenhouse gas emissions. Ratering and Schnell [44] revealed a widespread metabolic potential for nitrate reduction coupled to iron(II) oxidation among *Proteobacteria* in paddy soils. The families *Gallionellaceae*, *Comamonadaceae* and the order *Rhodospirillales* belonging to the *Proteobacteria* were enriched in the high N treatment on day 37 (Fig. SI 23). *Gallionellaceae* are typical microaerophilic Fe(II)-oxidizing microorganisms [73], yet some members are related to lithoautotrophic nitrate-reducing, Fe(II)-oxidizing microorganisms that have been successfully enriched in microbial cultures [[74], [75], [76], [77], [78]]. In paddy soils, members of the family *Gallionellaceae* were identified as potential key players for microbial nitrate reduction coupled to Fe(II) oxidation [79,80]. *Comamonadaceae* were found to be important decomposers in paddy soils [81] and are typically involved in the N-cycle mainly performing denitrification, which could potentially be linked to arsenite or Fe(II) oxidation [[82], [83], [84]]. *Rhodospirillales* are considered to play a role in N_2_O reduction [85]. These results highlight that N fertilization changes the microbial community and favors N-cycling microorganisms.

### 3.5 Implications of nitrogen fertilization in paddy soils for arsenic mobility and greenhouse gas emissions

Our results showed that nitrate reduction coupled to Fe(II) oxidation was stimulated by the addition of N fertilizer to the paddy soil. This (1) led to the formation of Fe(III) minerals immobilizing a maximum of 28 μg L^−1^ of dissolved arsenic and (2) prevented the reductive dissolution of Fe(III) minerals and the simultaneous release of arsenic from Fe mineral phases into solution, which was most successful under the highest N fertilizer application. The formation of Fe(III) (oxyhydr)oxides was likely more important for arsenic immobilization than As(III) oxidation. This is supported by increasing Fe(III) levels, decreasing As(III) levels, a higher abundance of Fe(II)-oxidizers, and the lack of a significant increase in *aioA* gene abundance in the high N treatment. Although such high concentrations may not be directly applied to paddy soils as the common drinking water limit (50 mg L^−1^) [86] for nitrate would be mostly exceeded, our data shows that constant nitrate concentrations, which could also be achieved by other methods such as applying slow release N fertilizer or different fertilization frequencies and quantities, could suppress Fe(III) reduction and prevent the mobilization of arsenic and limit methane emissions by suppression of methanogenesis. Whether the same mechanisms hold true if other common fertilizers, e.g., urea, ammonia, are applied to different paddy fields has to be investigated.

Minimizing arsenic concentrations in the porewater, in the rice plant and in the rice grain might become even more important in the future to counteract decreases in rice yield [19]. As it was shown that arsenic sequestration by Fe plaque or minerals is dependent on the growth stage of the rice plant, which is also critical for arsenic uptake by rice plants [57], our results could provide guidance for practical application. We showed that arsenic immobilization can occur quickly within just a few days and even long-term over 129 days, when KNO_3_ was applied at higher concentrations. Thus, the frequency and amount of N fertilizer application becomes more important in future rice cultivation as it has a great potential in minimizing arsenic concentrations. Furthermore, the cultivation of microbial key players, such as *Gallionellaceae*, from paddy soils would enable us to study nitrate reduction coupled to Fe(II) oxidation in more detail with the goal to identify parameters that control rates of Fe(II) oxidation and nitrate reduction, and as a consequence, N_2_O emissions and arsenic mobility.

In summary, our results showed that the highest N fertilizer application rate was most effective in retaining arsenic on Fe mineral phases and preventing the mobilization into solution, without increasing total greenhouse gas emissions compared to lower fertilizer application rates. These findings have enhanced our insight into how N fertilizer application influences the interconnected processes of the microbial Fe, N, and As cycles in paddy soils.

## Data availability statement

Raw sequencing data was deposited into the Sequence Read Archive (SRA) at NCBI under the BioProject accession number PRJNA922084 (https://www.ncbi.nlm.nih.gov/bioproject/PRJNA922084). The dataset supporting the findings of this study is published on Zenodo and can be accessed via https://doi.org/10.5281/zenodo.10679656.

## Funding sources

Deutsche Forschungsgemeinschaft (DFG, German Research Foundation, project ID 431072007).

## CRediT authorship contribution statement

**Hanna Grimm:** Conceptualization, Methodology, Validation, Formal analysis, Investigation, Data curation, Visualization, Writing – Original Draft, Writing – review & editing. **Soeren Drabesch:** Formal analysis, Writing – review & editing. **Alan Nicol:** Formal analysis, Writing – review & editing. **Daniel Straub:** Formal analysis, Data curation, Writing – review & editing. **Prachi Joshi:** Validation, Writing – review & editing. **Christiane Zarfl:** Conceptualization, Resources, Supervision, Project administration, Funding acquisition, Writing – review & editing. **Britta Planer-Friedrich:** Validation, Writing – review & editing. **E. Marie Muehe:** Validation, Supervision, Writing – review & editing. **Andreas Kappler:** Conceptualization, Resources, Supervision, Project administration, Funding acquisition, Writing – review & editing.

## Declaration of competing interest

The authors declare that they have no known competing financial interests or personal relationships that could have appeared to influence the work reported in this paper.

## References

[1] van Dijk M., Morley T., Rau M.L., Saghai Y. (2021). A meta-analysis of projected global food demand and population at risk of hunger for the period 2010–2050. Nature Food.

[2] Zhao X., Pu C., Ma S.-T., Liu S.-L., Xue J.-F., Wang X., Wang Y.-Q., Li S.-S., Lal R., Chen F., Zhang H.-L. (2019). Management-induced greenhouse gases emission mitigation in global rice production. Sci. Total Environ..

[3] Feng J., Chen C., Zhang Y., Song Z., Deng A., Zheng C., Zhang W. (2013). Impacts of cropping practices on yield-scaled greenhouse gas emissions from rice fields in China: a meta-analysis. Agric. Ecosyst. Environ..

[4] Liu Y., Ge T., van Groenigen K.J., Yang Y., Wang P., Cheng K., Zhu Z., Wang J., Li Y., Guggenberger G., Sardans J., Penuelas J., Wu J., Kuzyakov Y. (2021). Rice paddy soils are a quantitatively important carbon store according to a global synthesis. Communications Earth & Environment.

[5] Carlson K.M., Gerber J.S., Mueller N.D., Herrero M., MacDonald G.K., Brauman K.A., Havlik P., O'Connell C.S., Johnson J.A., Saatchi S., West P.C. (2017). Greenhouse gas emissions intensity of global croplands. Nat. Clim. Change.

[6] IPCC (2021).

[7] Korom S.F. (1992). Natural denitrification in the saturated zone A review. Water Resour. Res..

[8] Kelso B., Smith R.V., Laughlin R.J., Lennox S.D. (1997). Dissimilatory nitrate reduction in anaerobic sediments leading to river nitrite accumulation. Appl. Environ. Microbiol..

[9] Bleyen N., Smets S., Small J., Moors H., Leys N., Albrecht A., Cannière P. de, Schwyn B., Wittebroodt C., Valcke E. (2017). Impact of the electron donor on in situ microbial nitrate reduction in Opalinus Clay: results from the Mont Terri rock laboratory (Switzerland). Swiss J. Geosci..

[10] Kappler A., Bryce C., Mansor M., Lueder U., Byrne J.M., Swanner E.D. (2021). An evolving view on biogeochemical cycling of iron. Nat. Rev. Microbiol..

[11] Wang M., Hu R., Ruser R., Schmidt C., Kappler A. (2020). Role of chemodenitrification for N2O emissions from nitrate reduction in rice paddy soils. ACS Earth Space Chem..

[12] Hohmann C., Winkler E., Morin G., Kappler A. (2010). Anaerobic Fe(II)-oxidizing bacteria show as resistance and immobilize as during Fe(III) mineral precipitation. Environ. Sci. Technol..

[13] Hohmann C., Morin G., Ona-Nguema G., Guigner J.-M., Brown G.E., Kappler A. (2011). Molecular-level modes of as binding to Fe(III) (oxyhydr)oxides precipitated by the anaerobic nitrate-reducing Fe(II)-oxidizing Acidovorax sp. strain BoFeN1. Geochem. Cosmochim. Acta.

[14] Majumder S., Banik P. (2019). Geographical variation of arsenic distribution in paddy soil, rice and rice-based products: a meta-analytic approach and implications to human health. J. Environ. Manag..

[15] Muehe E.M., Kappler A. (2014). Arsenic mobility and toxicity in South and South-east Asia – a review on biogeochemistry, health and socio-economic effects, remediation and risk predictions. Environ. Chem..

[16] Ponnamperuma F.N. (1972). The chemistry of submerged soils. Adv. Agron..

[17] Takahashi Y., Minamikawa R., Hattori K.H., Kurishima K., Kihou N., Yuita K. (2004). Arsenic behavior in paddy fields during the cycle of flooded and non-flooded periods. Environ. Sci. Technol..

[18] Zobrist J., Dowdle P.R., Davis J.A., Oremland R.S. (2000). Mobilization of arsenite by dissimilatory reduction of adsorbed arsenate. Environ. Sci. Technol..

[19] Muehe E.M., Wang T., Kerl C.F., Planer-Friedrich B., Fendorf S. (2019). Rice production threatened by coupled stresses of climate and soil arsenic. Nat. Commun..

[20] Liu L., Shen R.-L., Zhao Z.-Q., Ding L.-J., Cui H.-L., Li G., Yang Y.-P., Duan G.-L., Zhu Y.-G. (2022). How different nitrogen fertilizers affect arsenic mobility in paddy soil after straw incorporation?. J. Hazard Mater..

[21] Wang F., Zhang J., Zeng Y., Wang H., Zhao X., Chen Y., Deng H., Ge L., Dahlgren R.A., Gao H., Chen Z. (2023). Arsenic mobilization and nitrous oxide emission modulation by different nitrogen management strategies in flooded ammonia-enriched paddy soils. Pedosphere.

[22] Wang X., Liu T., Li F., Li B., Liu C. (2018). Effects of simultaneous application of ferrous iron and nitrate on arsenic accumulation in rice grown in contaminated paddy soil. ACS Earth Space Chem..

[23] Hussain M.M., Bibi I., Niazi N.K., Shahid M., Iqbal J., Shakoor M.B., Ahmad A., Shah N.S., Bhattacharya P., Mao K., Bundschuh J., Ok Y.S., Zhang H. (2021). Arsenic biogeochemical cycling in paddy soil-rice system: interaction with various factors, amendments and mineral nutrients. Sci. Total Environ..

[24] Wang M., Hu R., Zhao J., Kuzyakov Y., Liu S. (2016). Iron oxidation affects nitrous oxide emissions via donating electrons to denitrification in paddy soils. Geoderma.

[25] Furukawa Y., Inubushi K. (2004). Effect of application of iron materials on methane and nitrous oxide emissions from two types of paddy soils. Soil Sci. Plant Nutr..

[26] Bolyen E., Rideout J.R., Dillon M.R., Bokulich N.A., Abnet C.C., Al-Ghalith G.A., Alexander H., Alm E.J., Arumugam M., Asnicar F., Bai Y., Bisanz J.E., Bittinger K., Brejnrod A., Brislawn C.J., Brown C.T., Callahan B.J., Caraballo-Rodríguez A.M., Chase J., Cope E.K., Da Silva R., Diener C., Dorrestein P.C., Douglas G.M., Durall D.M., Duvallet C., Edwardson C.F., Ernst M., Estaki M., Fouquier J., Gauglitz J.M., Gibbons S.M., Gibson D.L., Gonzalez A., Gorlick K., Guo J., Hillmann B., Holmes S., Holste H., Huttenhower C., Huttley G.A., Janssen S., Jarmusch A.K., Jiang L., Kaehler B.D., Kang K.B., Keefe C.R., Keim P., Kelley S.T., Knights D., Koester I., Kosciolek T., Kreps J., Langille M.G.I., Lee J., Ley R., Liu Y.-X., Loftfield E., Lozupone C., Maher M., Marotz C., Martin B.D., McDonald D., McIver L.J., Melnik A.V., Metcalf J.L., Morgan S.C., Morton J.T., Naimey A.T., Navas-Molina J.A., Nothias L.F., Orchanian S.B., Pearson T., Peoples S.L., Petras D., Preuss M.L., Pruesse E., Rasmussen L.B., Rivers A., Robeson M.S., Rosenthal P., Segata N., Shaffer M., Shiffer A., Sinha R., Song S.J., Spear J.R., Swafford A.D., Thompson L.R., Torres P.J., Trinh P., Tripathi A., Turnbaugh P.J., Ul-Hasan S., van der Hooft J.J.J., Vargas F., Vázquez-Baeza Y., Vogtmann E., Hippel M. von, Walters W., Wan Y., Wang M., Warren J., Weber K.C., Williamson C.H.D., Willis A.D., Xu Z.Z., Zaneveld J.R., Zhang Y., Zhu Q., Knight R., Caporaso J.G. (2019). Reproducible, interactive, scalable and extensible microbiome data science using QIIME 2. Nat. Biotechnol..

[27] Zavattaro L., Romani M., Sacco D., Bassanino M., Grignani C. (2008). Fertilization management of paddy fields in Piedmont (NW Italy). Ital. J. Agron..

[28] Chen J., Huang Y., Tang Y. (2011). Quantifying economically and ecologically optimum nitrogen rates for rice production in south-eastern China. Agric. Ecosyst. Environ..

[29] Schaedler F., Kappler A., Schmidt C. (2018). A revised iron extraction protocol for environmental samples rich in nitrite and carbonate. Geomicrobiol. J..

[30] Lueder U., Maisch M., Laufer K., Jorgensen B.B., Kappler A., Schmidt C. (2020). Influence of physical perturbation on Fe(II) supply in coastal marine sediments. Environ. Sci. Technol..

[31] Cornell R.M., Schwertmann U. (2003).

[32] Heron G., Crouzet C., Bourg A.C., Christensen T.H. (1994). Speciation of Fe(II) and Fe(III) in contaminated aquifer sediments using chemical extraction techniques. Environ. Sci. Technol..

[33] Stookey L.L. (1970). Ferrozine-a new spectrophotometric reagent for iron. Anal. Chem..

[34] Yang Z., Yu Y., Hu R., Xu X., Xian J., Yang Y., Liu L., Cheng Z. (2020). Effect of rice straw and swine manure biochar on N2O emission from paddy soil. Sci. Rep..

[35] Lueders T., Manefield M., Friedrich M.W. (2004). Enhanced sensitivity of DNA- and rRNA-based stable isotope probing by fractionation and quantitative analysis of isopycnic centrifugation gradients. Environ. Microbiol..

[36] Parada A.E., Needham D.M., Fuhrman J.A. (2016). Every base matters: assessing small subunit rRNA primers for marine microbiomes with mock communities, time series and global field samples. Environ. Microbiol..

[37] Apprill A., McNally S., Parsons R., Weber L. (2015). Minor revision to V4 region SSU rRNA 806R gene primer greatly increases detection of SAR11 bacterioplankton. Aquat. Microb. Ecol..

[38] Ewels P.A., Peltzer A., Fillinger S., Patel H., Alneberg J., Wilm A., Garcia M.U., Di Tommaso P., Nahnsen S. (2020). The nf-core framework for community-curated bioinformatics pipelines. Nat. Biotechnol..

[39] Straub D., Blackwell N., Langarica-Fuentes A., Peltzer A., Nahnsen S., Kleindienst S. (2020). Interpretations of environmental microbial community studies are biased by the selected 16S rRNA (gene) amplicon sequencing pipeline. Front. Microbiol..

[40] Di Tommaso P., Chatzou M., Floden E.W., Barja P.P., Palumbo E., Notredame C. (2017). Nextflow enables reproducible computational workflows. Nat. Biotechnol..

[41] Kurtzer G.M., Sochat V., Bauer M.W. (2017). Singularity: Scientific containers for mobility of compute. PLoS One.

[42] Peiffer S., Kappler A., Haderlein S.B., Schmidt C., Byrne J.M., Kleindienst S., Vogt C., Richnow H.H., Obst M., Angenent L.T., Bryce C., McCammon C., Planer-Friedrich B. (2021). A biogeochemical–hydrological framework for the role of redox-active compounds in aquatic systems. Nat. Geosci..

[43] Chen N., Fu Q., Wu T., Cui P., Fang G., Liu C., Chen C., Liu G., Wang W., Wang D., Wang P., Zhou D. (2021). Active iron phases regulate the abiotic transformation of organic carbon during redox fluctuation cycles of paddy soil. Environ. Sci. Technol..

[44] Ratering S., Schnell S. (2001). Nitrate-dependent iron(II) oxidation in paddy soil. Environ. Microbiol..

[45] Li X., Zhang W., Liu T., Chen L., Chen P., Li F. (2016). Changes in the composition and diversity of microbial communities during anaerobic nitrate reduction and Fe(II) oxidation at circumneutral pH in paddy soil. Soil Biol. Biochem..

[46] Liu T., Chen D., Li X., Li F. (2019). Microbially mediated coupling of nitrate reduction and Fe(II) oxidation under anoxic conditions. FEMS Microbiol. Ecol..

[47] Chen X.-P., Zhu Y.-G., Hong M.-N., Kappler A., Xu Y.-X. (2008). Effects of different forms of nitrogen fertilizers on arsenic uptake by rice plants. Environ. Toxicol. Chem..

[48] Jakus N., Mellage A., Höschen C., Maisch M., Byrne J.M., Mueller C.W., Grathwohl P., Kappler A. (2021). Anaerobic neutrophilic pyrite oxidation by a chemolithoautotrophic nitrate-reducing iron(II)-oxidizing culture enriched from a fractured aquifer. Environ. Sci. Technol..

[49] Weber K.A., Picardal F.W., Roden E.E. (2001). Microbially catalyzed nitrate-dependent oxidation of biogenic solid-phase Fe(II) compounds. Environ. Sci. Technol..

[50] Shelobolina E., Xu H., Konishi H., Kukkadapu R., Wu T., Blöthe M., Roden E. (2012). Microbial lithotrophic oxidation of structural Fe(II) in biotite. Appl. Environ. Microbiol..

[51] Pantke C., Obst M., Benzerara K., Morin G., Ona-Nguema G., Dippon U., Kappler A. (2012). Green rust formation during Fe(II) oxidation by the nitrate-reducing Acidovorax sp. strain BoFeN1. Environ. Sci. Technol..

[52] Zhang Y., Xu B., Han J., Shi L. (2021). Effects of drying-rewetting cycles on ferrous iron-involved denitrification in paddy soils. Water.

[53] Wei Z., Jin K., Li C., Wu M., Shan J., Yan X. (2022). Environmental factors controlling dissimilatory nitrate reduction to ammonium in paddy soil. J. Soil Sci. Plant Nutr..

[54] Luo D., Meng X., Zheng N., Li Y., Yao H., Chapman S.J. (2021). The anaerobic oxidation of methane in paddy soil by ferric iron and nitrate, and the microbial communities involved. Sci. Total Environ..

[55] Kögel-Knabner I., Amelung W., Cao Z., Fiedler S., Frenzel P., Jahn R., Kalbitz K., Kölbl A., Schloter M. (2010). Biogeochemistry of paddy soils. Geoderma.

[56] Li Y., Ning J., Li Q., Li L., Bolan N.S., Singh B.P., Wang H. (2022). Effects of iron and nitrogen-coupled cycles on cadmium availability in acidic paddy soil from Southern China. J. Soils Sediments.

[57] Yu H.-Y., Wang X., Li F., Li B., Liu C., Wang Q., Lei J. (2017). Arsenic mobility and bioavailability in paddy soil under iron compound amendments at different growth stages of rice. Environ. Pollut..

[58] Zhang J., Zhou W., Liu B., He J., Shen Q., Zhao F.-J. (2015). Anaerobic arsenite oxidation by an autotrophic arsenite-oxidizing bacterium from an arsenic-contaminated paddy soil. Environ. Sci. Technol..

[59] Dixit S., Hering J.G. (2003). Comparison of arsenic(V) and arsenic(III) sorption onto iron oxide minerals: implications for arsenic mobility. Environ. Sci. Technol..

[60] Zheng Q., Tu S., Chen Y., Zhang H., Hartley W., Ye B., Ren L., Xiong J., Tan W., Kappler A., Hou J. (2023). Micropore sites in ferrihydrite are responsible for its higher affinity towards As(III) relative to As(V). Geochem. Cosmochim. Acta.

[61] Yamaguchi N., Ohkura T., Hikono A., Yamaguchi H., Hashimoto Y., Makino T. (2017). Effects of iron amendments on the speciation of arsenic in the rice rhizosphere after drainage. Soils.

[62] Hou A.X., Chen G.X., Wang Z.P., van Cleemput O., Patrick Jr, W. H (2000). Methane and nitrous oxide emissions from a rice field in relation to soil redox and microbiological processes. Soil Sci. Soc. Am. J..

[63] Qin H., Tang Y., Shen J., Wang C., Chen C., Yang J., Liu Y., Chen X., Li Y., Hou H. (2018). Abundance of transcripts of functional gene reflects the inverse relationship between CH4 and N2O emissions during mid-season drainage in acidic paddy soil. Biol. Fertil. Soils.

[64] Haroon M.F., Hu S., Shi Y., Imelfort M., Keller J., Hugenholtz P., Yuan Z., Tyson G.W. (2013). Anaerobic oxidation of methane coupled to nitrate reduction in a novel archaeal lineage. Nature.

[65] Fan L., Dippold M.A., Ge T., Wu J., Thiel V., Kuzyakov Y., Dorodnikov M. (2020). Anaerobic oxidation of methane in paddy soil: role of electron acceptors and fertilization in mitigating CH4 fluxes. Soil Biol. Biochem..

[66] Vaksmaa A., Lüke C., van Alen T., Valè G., Lupotto E., Jetten M.S.M., Ettwig K.F. (2016). Distribution and activity of the anaerobic methanotrophic community in a nitrogen-fertilized Italian paddy soil. FEMS (Fed. Eur. Microbiol. Soc.) Microbiol. Ecol..

[67] Rivett M.O., Buss S.R., Morgan P., Smith J.W.N., Bemment C.D. (2008). Nitrate attenuation in groundwater: a review of biogeochemical controlling processes. Water Res..

[68] van Groenigen J.W., Huygens D., Boeckx P., Kuyper T.W., Lubbers I.M., Rütting T., Groffman P.M. (2015). The soil N cycle: new insights and key challenges. SOIL.

[69] Feng M., Du Y., Li X., Li F., Qiao J., Chen G., Huang Y. (2023). Insight into universality and characteristics of nitrate reduction coupled with arsenic oxidation in different paddy soils. Sci. Total Environ..

[70] Chen W., Yu X., Huang J., Zhao W., Ju J., Ye J., Qin H., Long Y. (2022). The synergy of Fe(III) and NO2- drives the anaerobic oxidation of methane. Sci. Total Environ..

[71] Chun S.-J., Kim Y.-J., Cui Y., Nam K.-H. (2021). Ecological network analysis reveals distinctive microbial modules associated with heavy metal contamination of abandoned mine soils in Korea. Environ. Pollut..

[72] Martins P.D., Jong A. de, Lenstra W.K., van Helmond N.A.G.M., Slomp C.P., Jetten M.S.M., Welte C.U., Rasigraf O. (2020). Enrichment of novel Verrucomicrobia, Bacteroidetes and Krumholzibacteria in an oxygen-limited, methane- and iron-fed bioreactor inoculated with Bothnian Sea sediments. Microbiology (Road Town, V. I. (Br.)).

[73] Emerson D., Field E.K., Chertkov O., Davenport K.W., Goodwin L., Munk C., Nolan M., Woyke T. (2013). Comparative genomics of freshwater Fe-oxidizing bacteria: implications for physiology, ecology, and systematics. Front. Microbiol..

[74] Huang Y.-M., Jakus N., Straub D., Konstantinidis K.T., Blackwell N., Kappler A., Kleindienst S. (2022). 'Candidatus ferrigenium straubiae' sp. nov., 'Candidatus ferrigenium bremense' sp. nov., 'Candidatus ferrigenium altingense' sp. nov., are autotrophic Fe(II)-oxidizing bacteria of the family Gallionellaceae. Syst. Appl. Microbiol..

[75] Jakus N., Blackwell N., Straub D., Kappler A., Kleindienst S. (2021). Presence of Fe(II) and nitrate shapes aquifer-originating communities leading to an autotrophic enrichment dominated by an Fe(II)-oxidizing Gallionellaceae sp. FEMS (Fed. Eur. Microbiol. Soc.) Microbiol. Ecol..

[76] Huang Y.-M., Straub D., Kappler A., Smith N., Blackwell N., Kleindienst S. (2021). A novel enrichment culture highlights core features of microbial networks contributing to autotrophic Fe(II) oxidation coupled to nitrate reduction. Microb. Physiol..

[77] Jakus N., Blackwell N., Osenbrück K., Straub D., Byrne J.M., Wang Z., Glöckler D., Elsner M., Lueders T., Grathwohl P., Kleindienst S., Kappler A. (2021). Nitrate removal by a novel lithoautotrophic nitrate-reducing, iron(II)-oxidizing culture enriched from a pyrite-rich limestone aquifer. Appl. Environ. Microbiol..

[78] Huang Y.-M., Straub D., Blackwell N., Kappler A., Kleindienst S. (2021). Meta-omics reveal Gallionellaceae and Rhodanobacter species as interdependent key players for Fe(II) oxidation and nitrate reduction in the autotrophic enrichment culture KS. Appl. Environ. Microbiol..

[79] Watanabe T., Katayanagi N., Agbisit R., Llorca L., Hosen Y., Asakawa S. (2021). Influence of alternate wetting and drying water-saving irrigation practice on the dynamics of Gallionella-related iron-oxidizing bacterial community in paddy field soil. Soil Biol. Biochem..

[80] Naruse T., Ban Y., Yoshida T., Kato T., Namikawa M., Takahashi T., Nishida M., Asakawa S., Watanabe T. (2019). Community structure of microaerophilic iron-oxidizing bacteria in Japanese paddy field soils. Soil Sci. Plant Nutr..

[81] Lu Y., Rosencrantz D., Liesack W., Conrad R. (2006). Structure and activity of bacterial community inhabiting rice roots and the rhizosphere. Environ. Microbiol..

[82] Sun W., Sierra-Alvarez R., Milner L., Oremland R., Field J.A. (2009). Arsenite and ferrous iron oxidation linked to chemolithotrophic denitrification for the immobilization of arsenic in anoxic environments. Environ. Sci. Technol..

[83] Bao P., Li G.-X. (2017). Sulfur-driven iron reduction coupled to anaerobic ammonium oxidation. Environ. Sci. Technol..

[84] Kappler A., Schink B., Newman D.K. (2005). Fe(III) mineral formation and cell encrustation by the nitrate-dependent Fe(II)-oxidizer strain BoFeN1. Geobiology.

[85] Ishii S., Ohno H., Tsuboi M., Otsuka S., Senoo K. (2011). Identification and isolation of active N2O reducers in rice paddy soil. ISME J..

[86] World Health Organization (2017). Fourth Edition Incorporating the First Addendum.

