# Supplementary material for: Arsenic immobilization and greenhouse gas emission depend on quantity and frequency of nitrogen fertilization in paddy soil
Source: Heliyon. 2024 Aug 3;10(16):e35706. doi: 10.1016/j.heliyon.2024.e35706 (PMC11379618; doi:10.1016/j.heliyon.2024.e35706)
Supplement: Multimedia component 1 [file mmc1.pdf]

Supporting information for

Arsenic immobilization and greenhouse gas emission depend on quantity and frequency of nitrogen fertilization in paddy soil

Hanna Grimm[1], Soeren Drabesch[1,2,3], Alan Nicol[4], Daniel Straub[5], Prachi Joshi[1], Christiane Zarfl[6], Britta Planer-Friedrich[4], E. Marie Muehe[2,3], Andreas Kappler[1,7*]

[1]*Geomicrobiology, Department of Geosciences, University of Tübingen, Schnarrenbergstrasse 94-96, 72076 Tübingen, Germany*

[2]*Plant Biogeochemistry, Department of Applied Microbial Ecology, Helmholtz Centre for Environmental Research - UFZ, Permoserstrasse 15, 04318 Leipzig, Germany*

[3]*Plant Biogeochemistry, Department of Geosciences, University of Tübingen, Schnarrenbergstrasse 94-96, 72076 Tübingen, Germany*

[4]*Environmental Geochemistry, Bayreuth Center for Ecology and Environmental Research (BayCEER), University of Bayreuth, Germany*

[5]*Quantitative Biology Center (QBiC), University of Tübingen, Germany*

[6]*Environmental Systems Analysis, Department of Geosciences, University of Tübingen, Schnarrenbergstrasse 94-96, 72076 Tübingen, Germany*

[7]*Cluster of Excellence: EXC 2124: Controlling Microbes to Fight Infection, Tübingen, Germany*

[*]Correspondence: andreas.kappler@uni-tuebingen.de

Table of Contents

Supplementary methods 3

Method S1: Soil characterization 3

Method S2: Microbial community analysis 5

Method S3: Pseudo-first-order kinetic model 6

Supplementary tables 7

Supplementary figures 13

References 33

Supplementary methods

Method S1: Soil characterization

Basic soil properties were analyzed in triplicates on soil samples after removal of plant debris and larger gravel. If not stated otherwise, analyses were performed at room temperature. For soil texture analysis, a soil dispersion was prepared by adding 25 mL of sodium pyrophosphate to 30 g of unsieved, fresh soil. After 30 min of stirring, the soil dispersion was filled up to a volume of 1000 mL and analyzed with a PARIO Soil Particle Analyzer (Meter Group, Germany).[1] After analysis, the soil was sieved (2 mm, 630 µm, 200 µm and 63 µm) to determine the sand and the fine fraction. Total loss of soil sample after sieving was below 5%. To determine the particle density, 20 g of dry soil was weighed into capillary pycnometer, filled up with deionized water and stepwise degassed before weight determination.[2] Bulk density was determined with a 100 cm^3 metal cylinder after drying fresh field soil at 105°C for 72 h.[3] Water contents from field fresh soil samples was determined by drying at 105°C for 72 h. Soil pH was determined by adding 10 mL of 0.01 M $CaCl_2$ to 2 g of field fresh soil samples (5:1, solution:soil) and measuring after 2 h and 24 h using a benchtop pH meter (SG2, Mettler-Toledo GmbH, Germany) equipped with a pH electrode (InLab Easy DIN, Mettler-Toledo GmbH, Germany).[4] The cation exchange capacity of the paddy soil was quantified in centrifuged samples after a 0.1 M $BaCl_2$ extraction (4 h, end-to-end shaker, 200 rpm), using 1.4 g of fresh soil (equivalent to ~1g of dry soil) and 25 mL of extraction solution, by microwave plasma atomic emission spectroscopy (4200 MP-AES, Agilent technologies, United States).[5] X-ray fluorescence (XRF) was used to determine total elemental contents of the dried paddy soil. To do so, glass beads were prepared by mixing 0.2333 g of dried and mortared sample with 3.9666 g of Fluxana FX-X65 (lithiumtetraborate:lithiummetaborate 66%:34%) and melted in a platinum crucible

using a Spetec Roto-Melt 2,0 μP at 0.45 for 6 min. Afterwards, the sample was poured into a platinum mold and loaded into a S8 Tiger (Bruker) prior to analysis. Total element concentrations were quantified using the calibration package GeoQuant (Bruker). The loss on ignition (LOI) was calculated as the percental weight difference between the dried (105°C for 24 h) and annealed sample powder (3000°C for 3 h) and is 6.06 wt%. The LOI and the total XRF sums add up to 99.93 wt%. Total soil carbon and nitrogen contents were analyzed for dry and mortared paddy soil samples by dry combustion (soliTOC cube, Elementar Analysensysteme GmbH, Germany). Water-extractable organic carbon and nitrogen species were determined in 0.45 μm filtered samples after extraction of 1 g dry weight soil with 5 mL of MQ (24 h, overhead shaker) by an elemental analyzer (multi N/C, 2100S, Analytik Jena GmbH) and segmented flow analysis (CFA, AutoAnalyzer 3, SEAL Analytical, Germany), respectively. To evaluate the presence of different Fe mineral phases and associated arsenic, sequential extractions were performed under anoxic conditions (rolling shaker or water bath). 1.3 g of fresh soil samples (equivalent to 1 g of dry soil) were extracted for 24 h with 10 mL of 1 M sodium acetate (pH 5, adjusted with acetic acid) targeting adsorbed Fe(II) and Fe in amorphous sulfide minerals (referred to as adsorbed Fe).[6,7] It is known that sodium acetate also extracts carbonates,[8] yet this is considered to play a minor role due to low total inorganic carbon contents and low pH (Table SI 1). This was followed by a 2 h extraction with 10 mL of 0.5 M HCl, extracting poorly crystalline Fe minerals and reduced Fe(II) minerals such as $FeCO_3$ and FeS (referred to as poorly crystalline Fe).[9] Lastly, samples were extracted for 24 h at 70°C with 10 mL of 6 M HCl for extraction of more crystalline Fe mineral phases and poorly reactive sheet silicate Fe or FeS species (referred to as crystalline Fe).[10]

Method S2: Microbial community analysis

Quantitative PCR (qPCR) was performed in technical triplicates for DNA and cDNA for bacterial 16S rRNA genes and for the *Geobacter* spp. marker gene. Due to low starting quantities of cDNA, only DNA analysis was performed for the functional genes *narG, nosZ, aioA* and *arrA*. A 7-fold standard dilution series was included in each qPCR assay. Data analysis was performed using the Bio-Rad CFX Maestro 1.1, software, version 4.1 (Bio-Rad, 2017). The qPCR primer sequences, gene-specific plasmid standards (pCR2.1®, Invitrogen, Darmstadt, Germany) and details of the thermal programs are given in Table SI 7. For 16S rRNA gene amplicon sequencing, Library preparation steps (Nextera, Illumina) and 250 bp paired-end sequencing with MiSeq (Illumina, San Diego, CA, USA) using v2 chemistry were performed by Microsynth AG (Balgach, Switzerland). Between 126,043 and 193,506 read pairs were obtained for each of the 6 samples (in total 930,001 read pairs). For data analysis, primers were trimmed, and untrimmed sequences were discarded (<6% per sample) with Cutadapt version 3.4.[11] Adapter and primer-free sequences were processed with DADA2 v1.22.0 to eliminate PhiX contamination, trim reads (before median quality drops below 35; forward reads were trimmed at 181 bp and reverse reads at 167 bp), correct errors, merge read pairs, and remove polymerase chain reaction (PCR) chimeras; ultimately, 6,891 amplicon sequencing variants (ASVs) were obtained across all samples.[12] Taxonomic classification was performed with DADA2 and the SILVA v138 database.[13] Intermediate results were imported into QIIME2 version 2021.8.0.[14] 208 ASVs classified as chloroplasts or mitochondria were removed, totaling <2% (average 0.86%) relative abundance per sample, and retaining 6,683 ASVs across all samples. Alpha rarefaction curves were produced with the QIIME2 diversity alpha-rarefaction plugin, which indicated that the richness of the samples had been fully observed.

Method S3: Pseudo-first-order kinetic model

Pseudo-first-order rate constants k for nitrate reduction were derived using eq. 1,

$$\ln\left(\frac{c_t}{c_0}\right) = -kt \quad (1)$$

with c_t: concentration of NO_3^- in mM at time t in days, c_0: initial concentrations of NO_3^- in mM, k: rate constant in day^{-1}. The model for pseudo-first-order kinetics was fitted to the experimental data with $y = \ln\left(\frac{c_t}{c_0}\right)$, $x = t$, where the slope corresponds to $-k$, fixing the y-intercept at 0. Half-life times were calculated using eq. 2,

$$t_{1/2} = \frac{\ln(2)}{k} \quad (2)$$

with $t_{1/2}$: half-life time in days and k: rate constant in day^{-1}.

Supplementary tables

Table SI 1 | Characterization of basic soil properties of paddy soil collected from Vercelli, Italy.

		Vercelli, Italy		
**Coordinates**		45°19'26" N, 8°22'25" E		
**Parent material**		River alluvium		
**Paddy management**		rice		
**N Fertilizer - Urea**	[kg N ha^{-1} $year^{-1}$]	279		
**Sampling depth**	[cm]	0-20		
**Soil texture**				
Sand	[%]	23.99	±	2.17
Silt	[%]	58.00	±	2.00
Clay	[%]	17.33	±	3.21
**CEC**	[cmol kg^{-1}]	5.45	±	0.04
**pH_{CaCl2}**		4.94	±	0.05
**Water content**	[%]	23.88	±	0.95
**TOC**	[%]	1.39	±	0.10
**TIC**	[%]	0.05	±	0.00
**TN**	[%]	0.12	±	0.00
**Adsorbed Fe***	[g kg^{-1}]	0.67	±	0.07
**Poorly crystalline Fe***	[g kg^{-1}]	2.23	±	0.18
**Crystalline Fe***	[g kg^{-1}]	18.03	±	3.58
**Total extractable Fe***	[g kg^{-1}]	20.93	±	3.58
**Adsorbed Fe-As***	[mg kg^{-1}]	3.90	±	0.55
**Poorly crystalline Fe-As***	[mg kg^{-1}]	0.63	±	0.03
**Crystalline Fe-As***	[mg kg^{-1}]	4.46	±	0.92
**Total extractable Fe-As***	[mg kg^{-1}]	8.99	±	1.07
**Na°**	[g kg^{-1}]	16.039		
**Mg°**	[g kg^{-1}]	17.212		
**Al°**	[g kg^{-1}]	72.351		
**Si°**	[g kg^{-1}]	302.73		
**P°**	[g kg^{-1}]	0.55		
**S°**	[g kg^{-1}]	0.48		
**K°**	[g kg^{-1}]	16.03		
**Ca°**	[g kg^{-1}]	17.27		
**Ti°**	[g kg^{-1}]	5.36		
**V°**	[g kg^{-1}]	0.07		
**Cr°**	[g kg^{-1}]	0.23		
**Mn°**	[g kg^{-1}]	0.49		
**Fe°**	[g kg^{-1}]	33.29		
**Ni°**	[g kg^{-1}]	0.24		
**Zn°**	[g kg^{-1}]	0.05		
**Zr°**	[g kg^{-1}]	0.16		
**Ba°**	[g kg^{-1}]	0.04		

Average and standard deviation are represented by triplicate measurements

*Obtained by sequentially extracting paddy soil samples with 1 M Na-acetate (adsorbed), 0.5 M HCl (poorly crystalline) and 6 M HCl (crystalline)

°Obtained by XRF analysis

Table SI 2 | Composition of artificial irrigation water used for microcosm incubation.

**Artificial groundwater composition**		
pH		6.2
electrical conductivity	µS cm^{-1}	550
$CaCl_2$	mmol L^{-1}	1.75
NaCl		0.44
$MgCl_2$		0.82
KCl		0.08

Table SI 3 | Volume of KNO_3 stock solution added to N fertilized microcosms.

**Treatment**	**KNO_3 stock**	**1st fertilization Volume added**	**2nd fertilization Volume added**
	mg N L^{-1}	mL	mL
Control	0	0	0
Low	777.81	0.83	0.86
Medium	2290.98	0.8	0.84
High	4610.98	0.8	0.83

Table SI 4 | Overview of ICP-MS parameters for total arsenic analysis from sequential extractions resembling Fe-bound arsenic.

**Element**	**Calibration range**	**Detection limit**	**Quality control 1**	**Quality control 2**	**Quality control 3**	**Instrument**
			ppb			
**Arsenic**	0-500	0.04	1.76 ± 0.24	2.40 ± 0.20	47.22 ± 2.55	Agilent 7900
		0.47	2.27 ± 0.5	66.75 ± 16.01		Agilent 7900

Table SI 5 | Recovery of total arsenic by comparing the sum of arsenite and arsenate obtained from arsenic speciation analysis with data from total arsenic analysis for representative samples.

Days	Treatment	Replicate	Arsenite	Arsenate	Total As (sum of species)	Total As (measured)	Recovery
			μg L^{-1}	μg L^{-1}	μg L^{-1}	μg L^{-1}	%
71	Control	2	27.03	26.99	54.02	54.94	98.33
71	Control	3	29.76	28.32	58.08	63.95	90.81
71	Low	3	31.42	25.58	57.00	59.65	95.54
85	Control	1	30.72	46.76	77.48	79.61	97.32
85	Control	2	39.81	43.14	82.95	92.60	89.57
85	Control	3	37.13	30.22	67.35	77.53	86.88
125	Control	1	30.11	31.53	61.64	64.53	95.52
125	Control	2	35.17	44.75	79.92	81.08	98.56
125	Control	3	31.06	27.65	58.71	58.63	100.14
125	Low	1	27.62	21.60	49.22	53.08	92.72
125	Low	3	38.62	30.53	69.15	72.92	94.83
129	Control	1	21.80	31.19	52.99	52.34	101.24
129	Control	2	26.70	37.28	63.98	67.37	94.98
129	Control	3	28.09	42.57	70.67	82.54	85.61
129	Low	1	27.78	26.67	54.46	51.76	105.22
129	Low	3	36.09	43.44	79.52	81.72	97.31

Recovery on average by 94.78±23.91%.

Table SI 6 | Calibration parameters of greenhouse gases CO_2, N_2O and CH_4.

Gas	Calibration range	Slope	Intercept	R^2	Detection limit	Quantification limit
	ppm				ppm	ppm
CO_2	0-200	34.98	-14.93	0.9985	-0.13	1.25
N_2O	0-3.5	26.18	0.01	0.9916	0.01	0.01
CH_4	0-2	26.87	-0.50	0.9902	0.08	0.29

Table SI 7 | Standards, primers and thermal profiles used in qPCR assays for different target genes.

Target gene	Standard	Primer	Primer sequence (5' -> 3')	Primer concentration (nM)	Thermal program	References
Bacterial 16S rRNA gene	*Thiomonas* sp.	515F	TCGTCGGCAGCGTCAGATGTGTATAAGAGACAGGTGYCAGCMGCCGCGGTA	250	95°C - 3'; (95°C - 10"; 55°C - 30") x 40; 95°C - 30"; 60-95°C - 5"	[15]
		806R	GTCTCGTGGGCTCGGAGATGTGTATAAGAGACAGGGACTACNVGGGTWTCTAAT	250		
Geobacter-aceae 16S rRNA gene	*Geobacter* sp.	Geo577F	GCGTGTAGGCGGTTTSTTAA	250	95°C - 3'; (95°C - 30"; 55°C - 20"; 72°C - 30") x 40; 95°C - 2'; 60-95°C - 5"	modified after Stults et al.[16]
		Geo822R	TACCCGCRACACCTAGTACT	250		
narG	*Pseudo-monas aeruginosa*	narG-F	TCGCCSATYCCGGCSATGTC	250	95°C - 3'; (95°C - 10"; 62°C - 20") x 40; 95°C - 2'; 70-95°C - 5"	[17]
		narG-R	GAGTTGTACCAGTCRGCSGAYTCSG	250		
nosZ	*Ensifer meliloti 1021*	nosZ2F	CGCRACGGCAASAAGGTSMSSGT	250	95°C - 3'; (95°C - 15"; 60°C - 25") x 40; 95°C - 2'; 65-95°C - 5"	[18]
		nosZ2R	CAKRTGCAKSGCRTGGCAGAA	250		
aioA	clone Red_G05 As SF	aioA-1F	TGCATCGTSGGBTGYGGNTA	500	95°C - 3'; (95°C - 30"; 60°C - 30") x 40; 95°C - 2'; 65-95°C - 5"	[19]
		aioA-1R	ACSACGCABTCYTTGTCSGG	500		
arrA	clone Red_A06 As SF	arrA-F	GGYSTGGGGCWSCGAYCC	500	95°C - 3'; (95°C - 30"; 62°C - 40") x 40; 95°C - 2'; 65-95°C - 5"	[20]
		arrA-R	GGMASCCASTYGTGGGMCTT	500		

Table SI 8 | Rate constants and coefficient of determination of pseudo-first-order reaction kinetics for nitrate reduction in low, medium and high N treatment.

Treatment	Fertilization period	Time	Rate constant *k*	half-life time	R^2
		days	$days^{-1}$	days	
Low	1st	0-10	0.40	1.73	0.9507
	2nd	49-55	0.75	0.92	0.9877
Medium	1st	0-16	0.23	2.98	0.8567
	2nd	49-71	0.24	2.91	0.9095
High	1st	0-37	0.08	8.54	0.8171
	2nd	49-129	0.05	12.96	0.8158

Table SI 9 | Proportion of reduced nitrate by oxidized dissolved iron(II) based on theoretical stoichiometry ratio of 1:5 ($nitrate_{red.}$:$iron(II)_{ox.}$) during autotrophic nitrate reduction coupled to iron(II) oxidation for the low, medium and high N treatment during the 1st and 2nd fertilization period. Reduced nitrate and oxidized Fe(II) was calculated as the difference between day 49 and 0 and between day 49 and 129 for the first and second fertilization period, respectively.

Fertilization	Treatment	Dissolved $nitrate_{red.}$	Dissolved $Fe(II)_{ox.}$	Contribution of dissolved $Fe(II)_{ox.}$ to $nitrate_{red.}$
		mM	mM	%
1st period	Low	0.17 ± 0.01	0.03 ± 0.01	3.62 ± 1.74
	medium	0.53 ± 0.02	0.12 ± 0.02	4.61 ± 0.75
	High	0.97 ± 0.09	0.14 ± 0.01	2.95 ± 0.39
2nd period	Low	0.45 ± 0.02	0.12 ± 0.01	5.55 ± 0.57
	medium	1.18 ± 0.15	0.25 ± 0.02	4.16 ± 0.68
	High	2.58 ± 0.05	0.06 ± 0.02	0.48 ± 0.13

Table SI 10 | Results of a Kruskal Wallis test to identify differences in total global warming potential (GWP), CO_2, CH_4 and N_2O emissions between treatments. The p-value indicates significant differences among treatments at the 0.05 significance level.

Comparison	χ^2	df	p-value
Treatment vs total GWP	7.35	3	0.06158
Treatment vs CO_2	8.32	3	0.03987
Treatment vs CH_4	9.73	3	0.02101
Treatment vs N_2O	8.8	3	0.03203

Table SI 11 | Results of the ANOVA (a) and the post-hoc test (Tukey test) (b) across different treatments. The p-value indicates significant differences among treatments at the 0.05 significance level.

a) ANOVA

Comparison	Sum of squares (SS)	Degrees of Freedom (DF)	Mean square (MS)	F-value	p-value
Treatment vs Proteobacteria	54.52	5	10.905	44.13	$2.58*10^{-7}$
Treatment vs Verrucomicrobiota	2.701	5	0.5402	4.257	0.0185
Treatment vs 16S rRNA transcript	658.5	3	219.51	11.07	0.0032

b) Tukey Test

Parameter	Group	Timepoint	p-value
Proteobacteria	Control vs High	day 37	$1.5*10^{-6}$
Verrucomicrobiota	Control vs High	day 37	0.0444
16S rRNA transcript number	Control vs High	day 37	0.0167

Supplementary figures

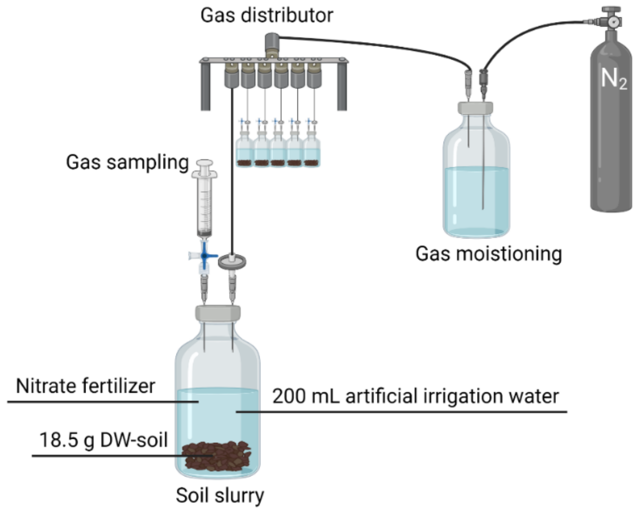

Figure SI 1 | Overview of experimental setup of microcosm experiment.

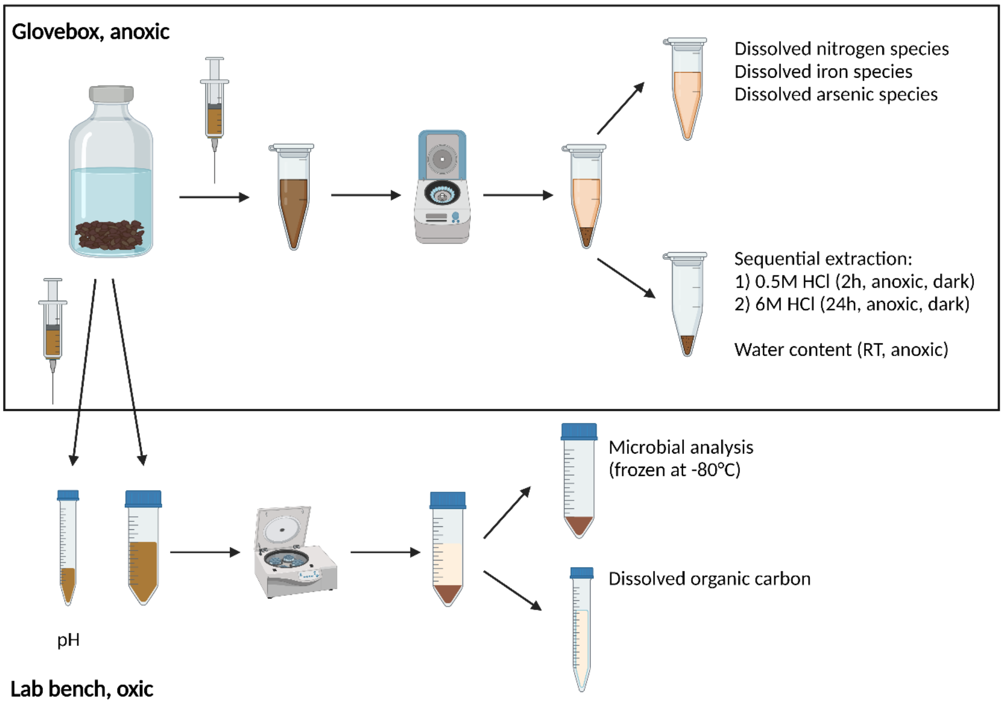

Figure SI 2 | Sampling overview, starting with a soil slurry sample taken in the glovebox for dissolved nitrogen species, Fe, arsenic and for water content. The dried soil was further sequentially extracted by 0.5 M HCl (2 h) and 6 M HCl (24 h). At selected timepoints a soil slurry sample was taken for pH, dissolved organic carbon and microbial analysis.

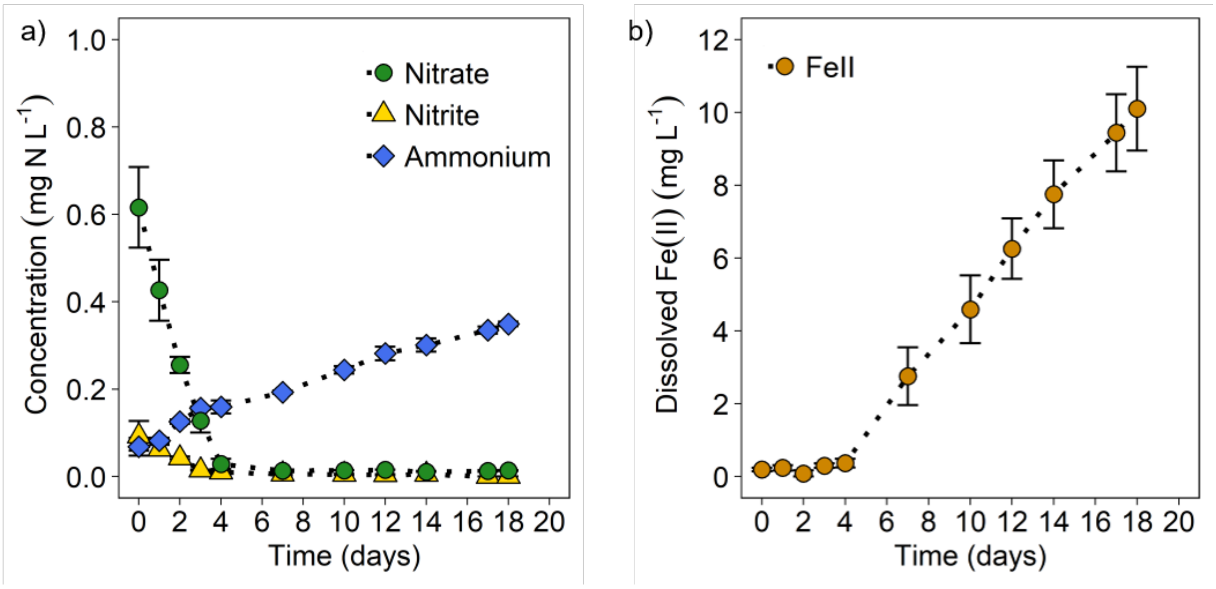

Figure SI 3 | Dissolved nitrate, nitrite and ammonium concentration in mg N L^{-1} (a) and dissolved Fe(II) in mg L^{-1} (b) during pre-incubation. Mean ± standard deviation is shown for biological triplicates.

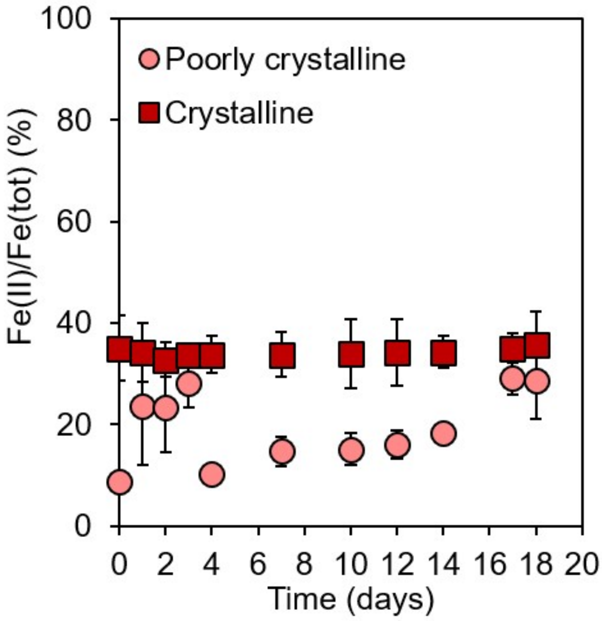

Figure SI 4 | Poorly crystalline and crystalline Fe(II)/Fe(tot) ratio in % during pre-incubation. Mean ± combined standard deviation from Fe(II) and Fe(tot) analysis is shown for biological triplicates.

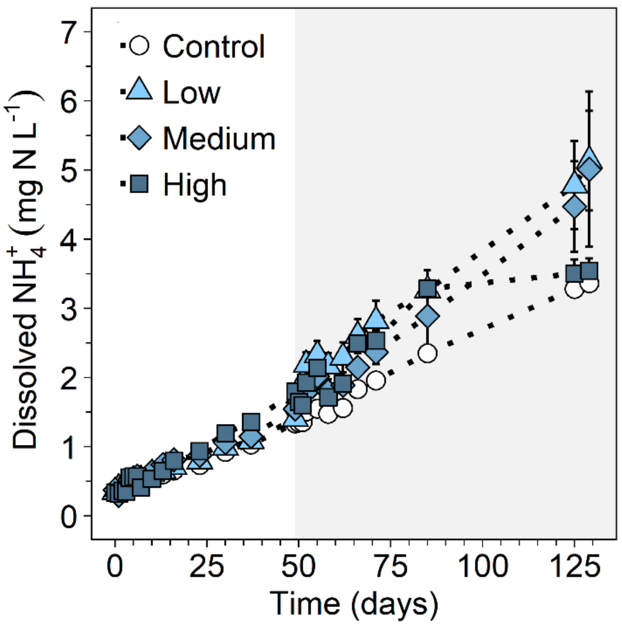

Figure SI 5 | Dissolved ammonium concentrations in mg N L^{-1} during the 129 days of incubation for the non-fertilized control and the N fertilized treatments (low N, medium N, high N). Mean ± standard deviation is shown for biological triplicates and the mean ± range for the low N treatment in the second fertilization for biological duplicates. The white background illustrates the first (0-49 days) and the grey background the second (49-129 days) nitrate fertilization period.

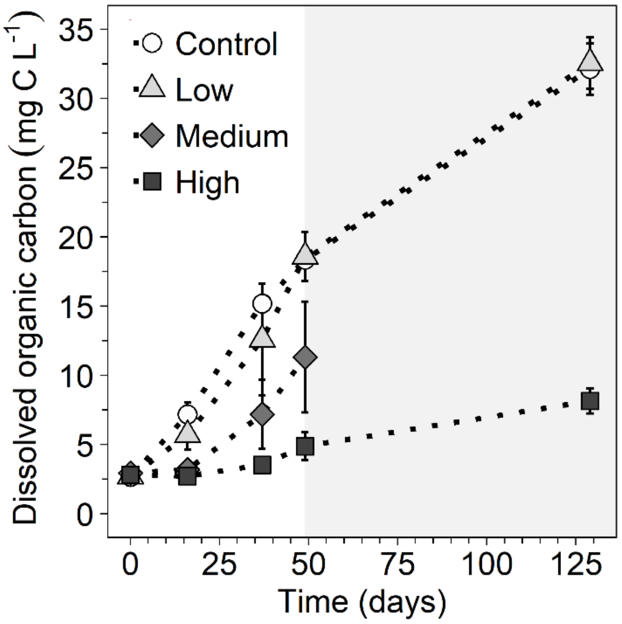

Figure SI 6 | Dissolved organic carbon in mg C L^{-1} during the 129 days of incubation for the non-fertilized control and the N fertilized treatments (low N, medium N, high N). Mean ± standard deviation is shown for biological triplicates and the mean ± range for the low N treatment in the second fertilization for biological duplicates. The white background illustrates the first (0-49 days) and the grey background the second (49-129 days) nitrate fertilization period. No measurement for dissolved organic carbon was possible on day 129 for the medium N treatment.

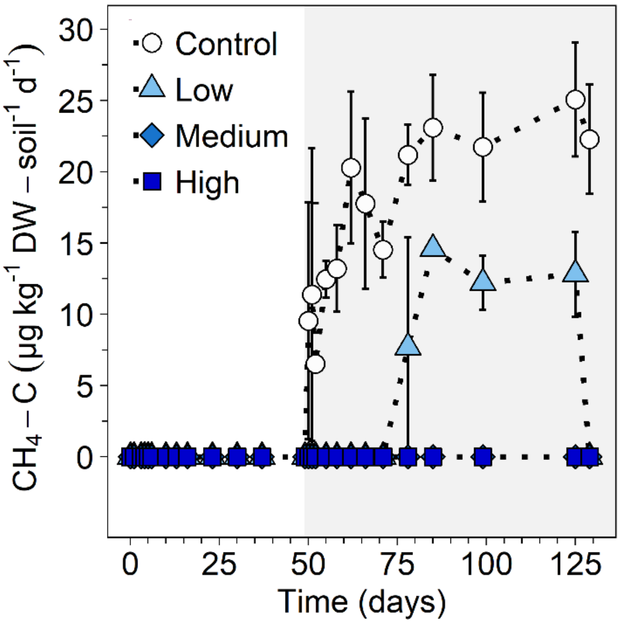

Figure SI 7 | Rates of CH_4-C emissions in µg kg^{-1} DW-soil^{-1} d^{-1} during the 129 days of incubation for the non-fertilized control and the N fertilized treatments (low N, medium N, high N). Mean ± standard deviation is shown for biological triplicates and the mean ± range for the low N treatment in the second fertilization for biological duplicates. The white background illustrates the first (0-49 days) and the grey background the second (49-129 days) nitrate fertilization period.

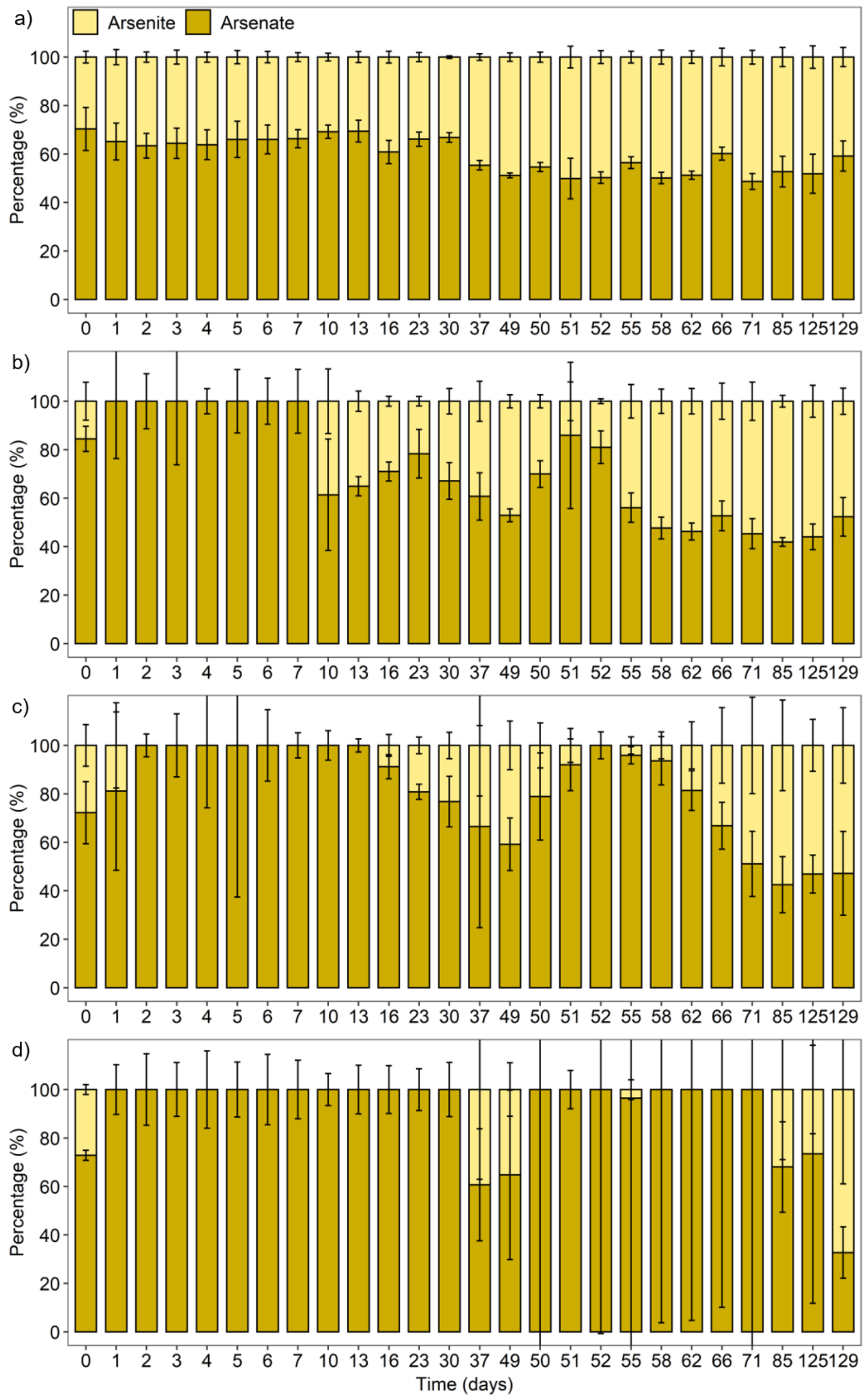

a)
Arsenite
Arsenate
Percentage (%)
b)
c)
d)
Time (days)

Figure SI 8 | Dissolved arsenite (dark) and arsenate (bright) percentages during the 129 days of incubation for the non-fertilized control (a), low N (b), medium N (c) and high N (d) fertilizer treatment. Mean ± standard deviation is shown for biological triplicates and the mean ± range for the low N treatment in the second fertilization for biological duplicates.

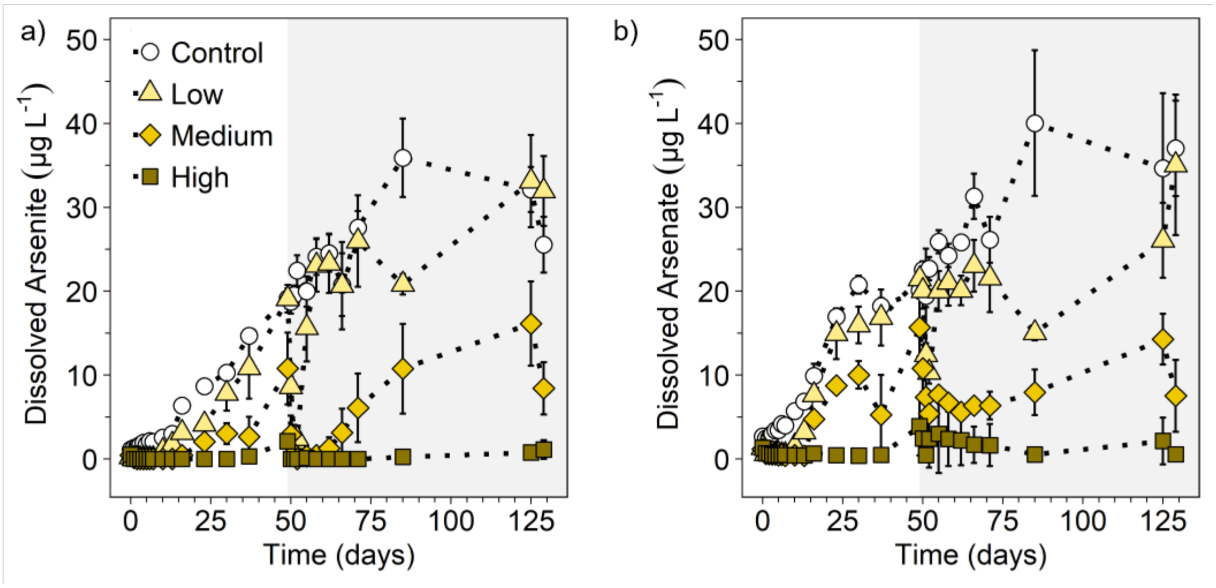

Figure SI 9 | Dissolved arsenite (a) and arsenate (b) concentrations in µg L^{-1} during the 129 days of incubation for the non-fertilized control and the N fertilized treatments (low N, medium N, high N). Mean ± standard deviation is shown for biological triplicates and the mean ± range for the low N treatment in the second fertilization for biological duplicates. The white background illustrates the first (0-49 days) and the grey background the second (49-129 days) nitrate fertilization period.

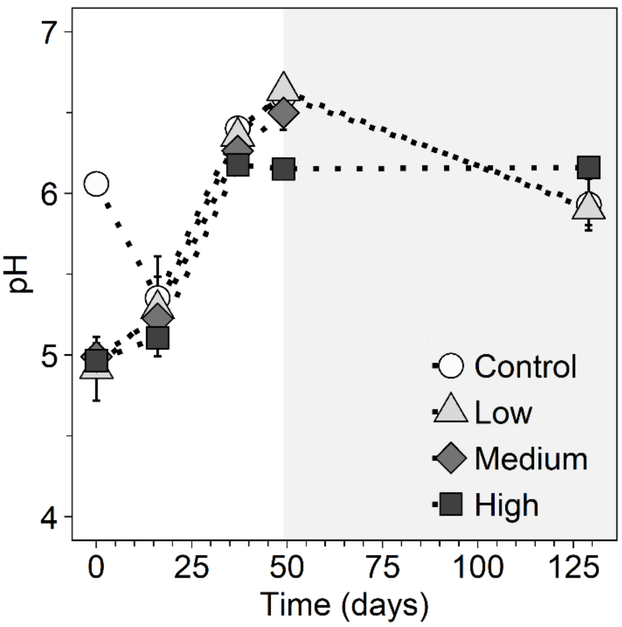

Figure SI 10 | pH of the soil slurry during the 129 days of incubation for the non-fertilized control and the N fertilized treatments (low N, medium N, high N). Mean ± standard deviation is shown for biological triplicates and the mean ± range for the low N treatment in the second fertilization for biological duplicates. The white background illustrates the first (0-49 days) and the grey background the second (49-129 days) nitrate fertilization period. No measurement for pH was possible on day 129 for the medium N treatment. Note that the y-axis spans pH values from 4 to 7.

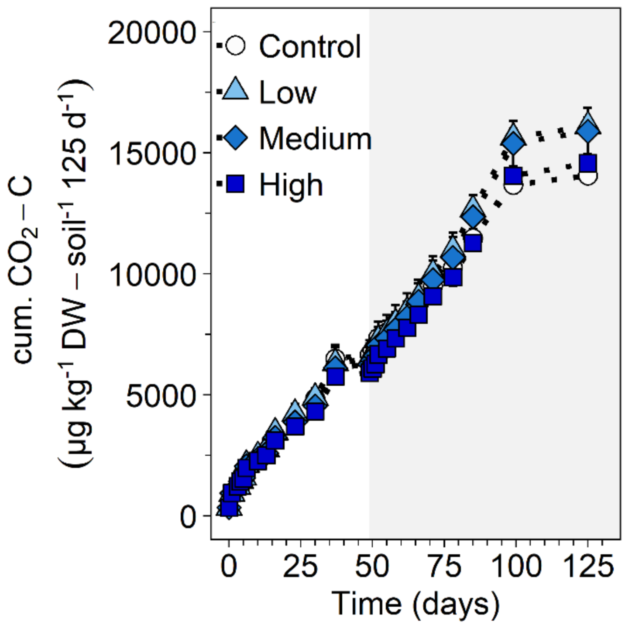

Figure SI 11 | Cumulative CO_2-C in µg kg^{-1} DW-$soil^{-1}$ 125 $days^{-1}$ during the 129 days of incubation for the non-fertilized control and the N fertilized treatments (low N, medium N, high N). Mean ± standard deviation is shown for biological triplicates and the mean ± range for the low N treatment in the second fertilization for biological duplicates. The white background illustrates the first (0-49 days) and the grey background the second (49-129 days) nitrate fertilization period.

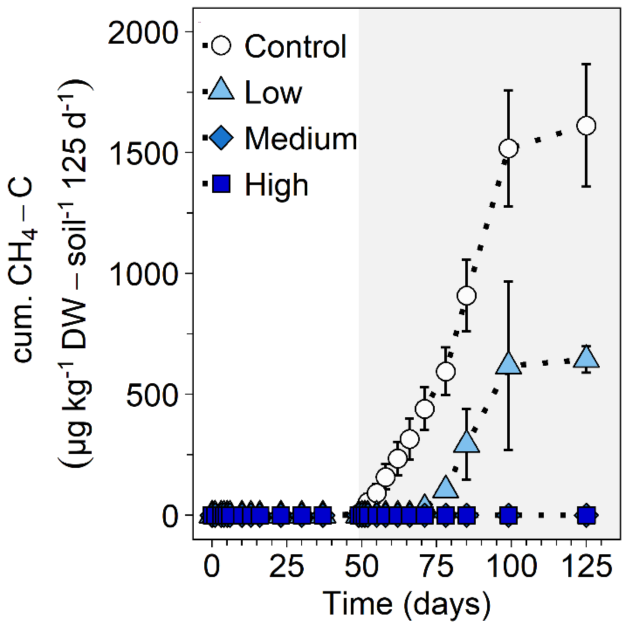

Figure SI 12 | Cumulative CH_4-C in µg kg^{-1} DW-soil^{-1} 125 days^{-1} during the 129 days of incubation for the non-fertilized control and the N fertilized treatments (low N, medium N, high N). Mean ± standard deviation is shown for biological triplicates and the mean ± range for the low N treatment in the second fertilization for biological duplicates. The white background illustrates the first (0-49 days) and the grey background the second (49-129 days) nitrate fertilization period.

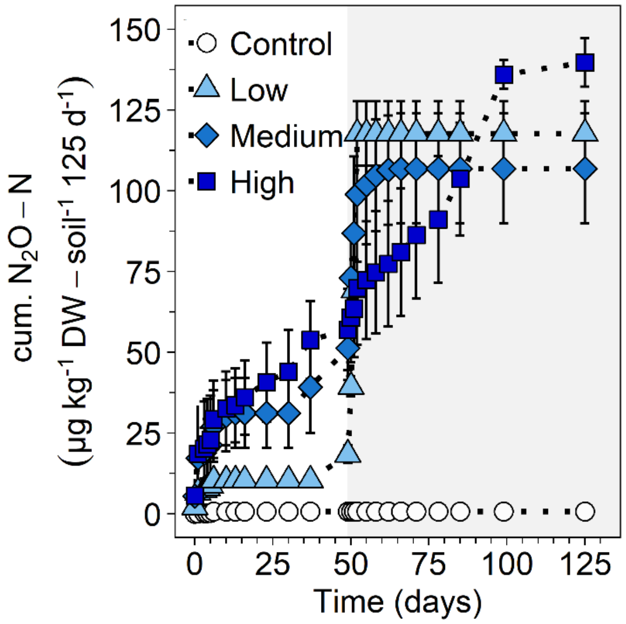

Figure SI 13 | Cumulative N_2O-N in µg kg^{-1} DW-$soil^{-1}$ 125 $days^{-1}$ during the 129 days of incubation for the non-fertilized control and the N fertilized treatments (low N, medium N, high N). Mean ± standard deviation is shown for biological triplicates and the mean ± range for the low N treatment in the second fertilization for biological duplicates. The white background illustrates the first (0-49 days) and the grey background the second (49-129 days) nitrate fertilization period.

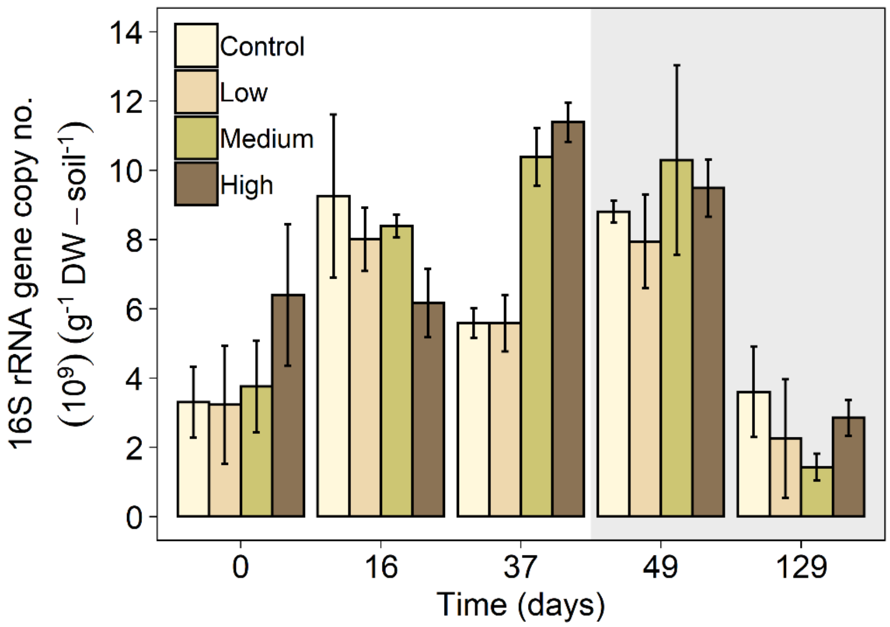

Figure SI 14 | 16S rRNA gene copy numbers (10^9) in g^{-1} DW-soil^{-1} at different timepoints during the 129 days of incubation for the non-fertilized control and the N fertilized treatments (low N, medium N, high N). Mean ± standard deviation is shown for biological triplicates and the mean ± range for the low N treatment in the second fertilization for biological duplicates. The white background illustrates the first (0, 16, 37 days) and the grey background the second (49, 129 days) nitrate fertilization period.

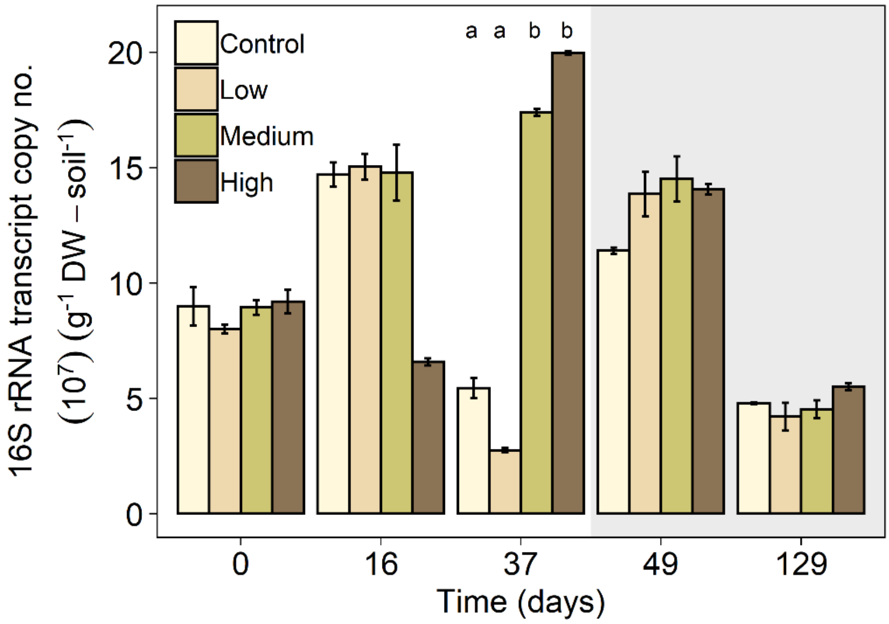

Figure SI 15 | 16S rRNA transcript copy numbers (10^7) in g^{-1} DW-soil^{-1} at different timepoints during the 129 days of incubation for the non-fertilized control and the N fertilized treatments (low N, medium N, high N). Mean ± standard deviation is shown for biological triplicates and the mean ± range for the low N treatment in the second fertilization for biological duplicates. Small letters indicate significant differences on day 37 between treatments ($p<0.05$, one-way ANOVA). The white background illustrates the first (0, 16, 37 days) and the grey background the second (49, 129 days) nitrate fertilization period.

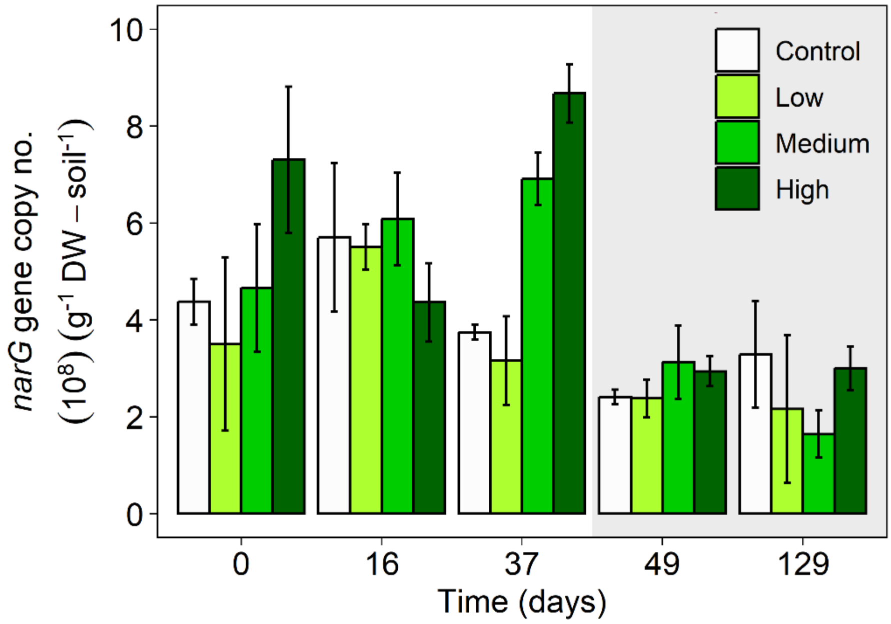

Figure SI 16 | Quantifying the potential for nitrate reduction with *narG* gene copy numbers (10^8) in g^{-1} DW-soil^{-1} responsible for nitrate reduction at different timepoints during the 129 days of incubation for the non-fertilized control and the N fertilized treatments (low N, medium N, high N). The white background illustrates the first (0, 16, 37 days) and the grey background the second (49, 129 days) nitrate fertilization period.

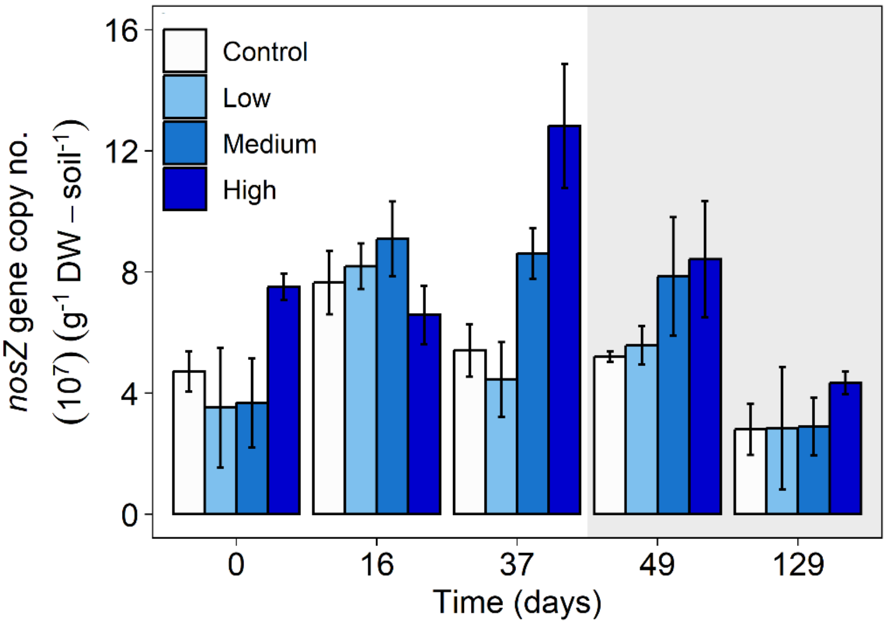

Figure SI 17 | Quantifying the potential for nitrous oxide reduction with *nosZ* gene copy numbers (10^7) in g^{-1} DW-soil^{-1} at different timepoints during the 129 days of incubation for the non-fertilized control and the N fertilized treatments (low N, medium N, high N). The white background illustrates the first (0, 16, 37 days) and the grey background the second (49, 129 days) nitrate fertilization period.

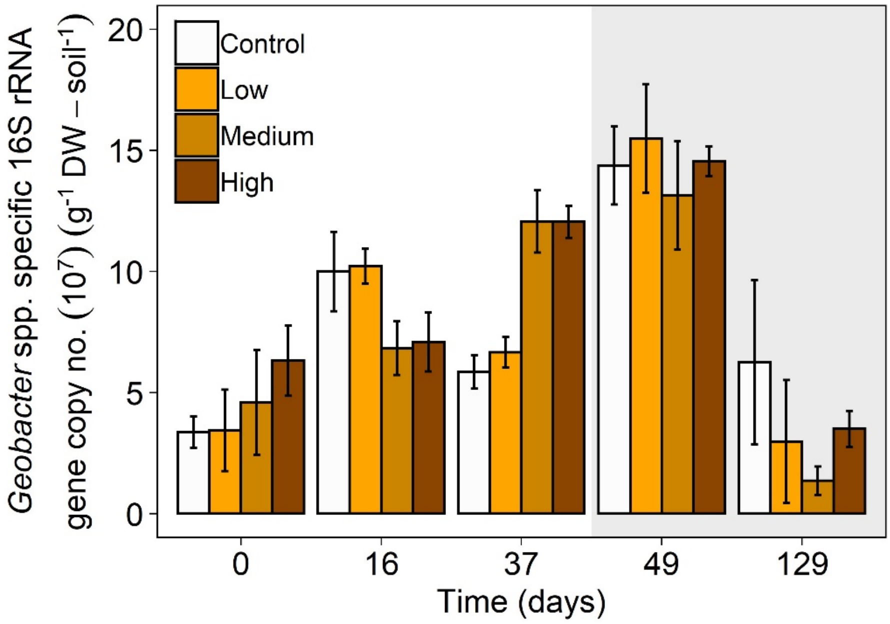

Figure SI 18 | Fe(III)-reducing bacterial numbers approximated by *Geobacter* spp. specific 16S rRNA gene copy numbers (10^7) in g^{-1} DW-soil^{-1} at different timepoints during the 129 days of incubation for the non-fertilized control and the N fertilized treatments (low N, medium N, high N). The white background illustrates the first (0, 16, 37 days) and the grey background the second (49, 129 days) nitrate fertilization period.

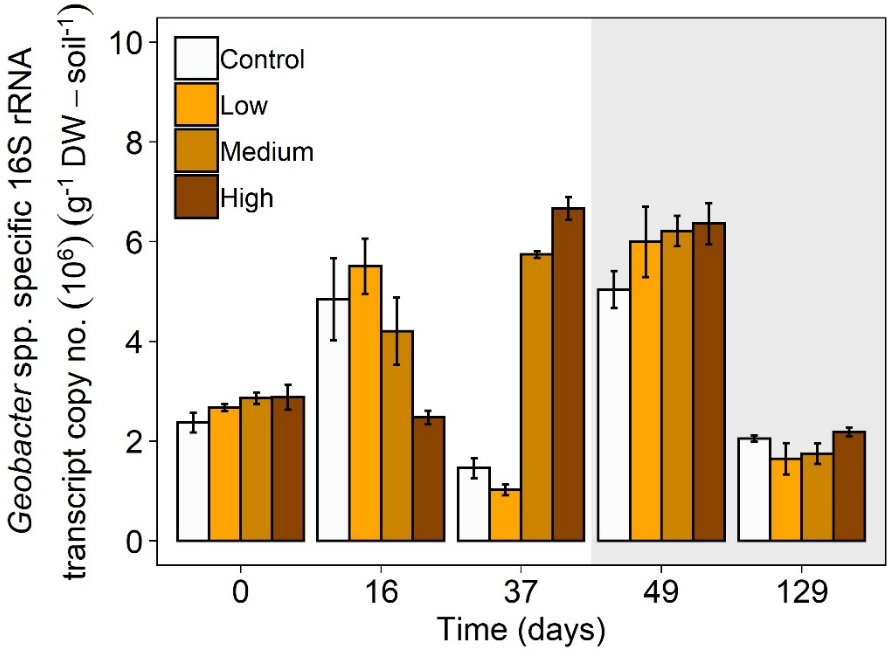

Figure SI 19 | Fe(III)-reducing bacterial activity approximated by *Geobacter* spp. specific 16S rRNA transcript numbers (10^6) in g^{-1} DW-soil^{-1} at different timepoints during the 129 days of incubation for the non-fertilized control and the N fertilized treatments (low N, medium N, high N). The white background illustrates the first (0, 16, 37 days) and the grey background the second (49, 129 days) nitrate fertilization period.

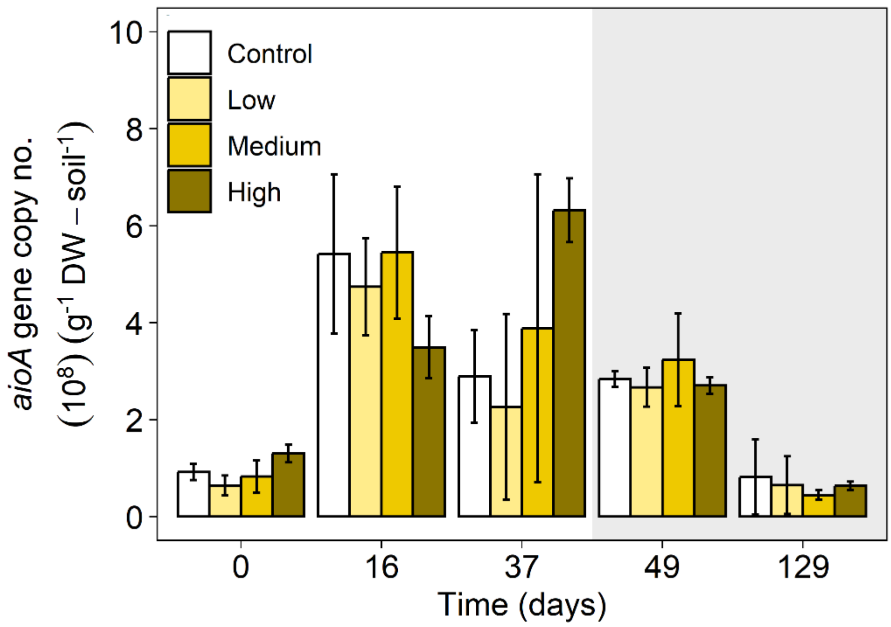

Figure SI 20 | Potential for arsenite oxidation quantified by *aioA* gene copy numbers (10^8) in g^{-1} DW-soil^{-1} at different timepoints during the 129 days of incubation for the non-fertilized control and the N fertilized treatments (low N, medium N, high N). The white background illustrates the first (0, 16, 37 days) and the grey background the second (49, 129 days) nitrate fertilization period.

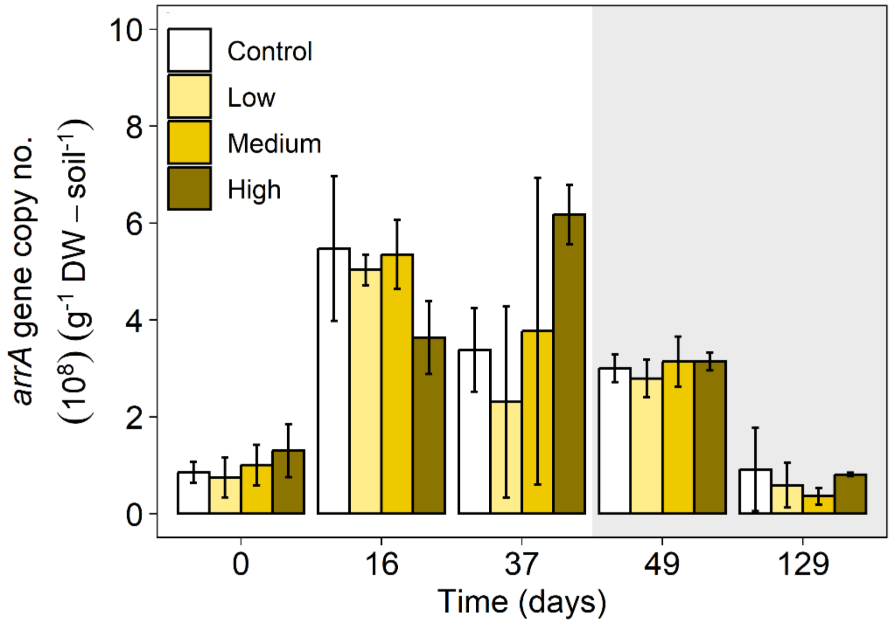

Figure SI 21 | Potential for arsenate reduction quantified by *arrA* gene copy numbers (10^8) in g^{-1} DW-soil^{-1} at different timepoints during the 129 days of incubation for the non-fertilized control and the N fertilized treatments (low N, medium N, high N). The white background illustrates the first (0, 16, 37 days) and the grey background the second (49, 129 days) nitrate fertilization period.

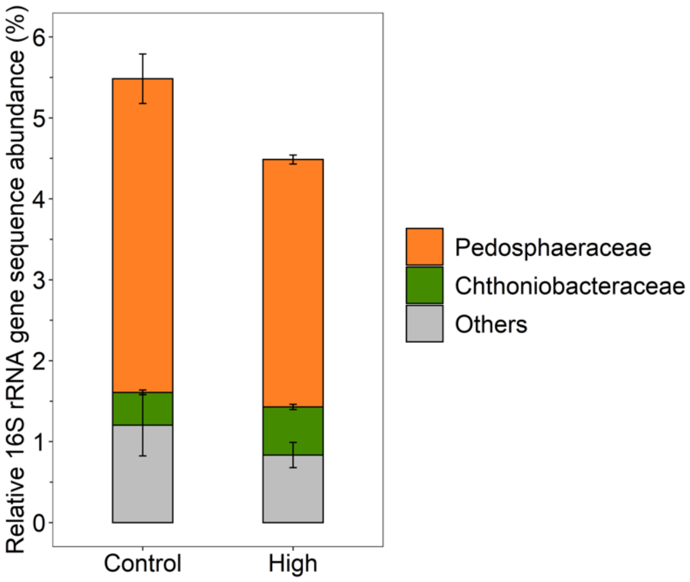

Figure SI 22 | Relative 16S rRNA gene sequence abundance in % on family level of the phyla *Verrucomicrobiota* for the control and high N treatment on day 37. “Others” represent families with abundances below 0.5% on average.

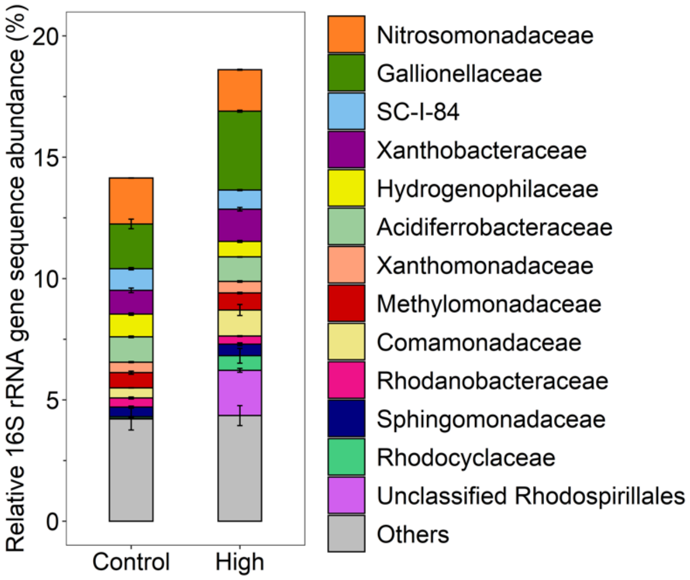

Figure SI 23 | Relative 16S rRNA gene sequence abundance in % on family level of the phyla *Proteobacteria* for the control and high N treatment on day 37. “Others” represent families with abundances below 0.5% on average.

References

(1) DIN 18123. Soil, investigation and testing - Determination of grain-size distribution **2011**.

(2) DIN 18124. Soil, investigation and testing - Determination of density of solid particles - Capillary pyknometer, wide mouth pycnometer, gas pycnometer **2011**.

(3) DIN EN ISO 17828. Solid biofuels - Determination of bulk density **2016**.

(4) ISO 10390. Soil, treated biowaste and sludge - Determination of pH **2021**.

(5) Muehe, E. M.; Wang, T.; Kerl, C. F.; Planer-Friedrich, B.; Fendorf, S. Rice production threatened by coupled stresses of climate and soil arsenic. *Nature communications* **2019**, *10* (1), 4985. DOI: 10.1038/s41467-019-12946-4.

(6) Roden, E. E.; Zachara, J. M. Microbial Reduction of Crystalline Iron(III) Oxides: Influence of Oxide Surface Area and Potential for Cell Growth. *Environmental Science & Technology* **1996**, *30* (5), 1618–1628. DOI: 10.1021/es9506216.

(7) Shannon, R. D.; White, J. R. The selectivity of a sequential extraction procedure for the determination of iron oxyhydroxides andiron sulfides in lake sediments. *Biogeochemistry* **1991**, *14* (3), 193–208. DOI: 10.1007/BF00000807.

(8) A. Tessier; P. G. C. Campbell; M. Bisson. Sequential extraction procedure for the speciation of particulate trace metals. *Analytical Chemistry* **1979**, *51* (7), 844–851.

(9) Heron, G.; Crouzet, C.; Bourg, A. C.; Christensen, T. H. Speciation of Fe(II) and Fe(III) in Contaminated Aquifer Sediments Using Chemical Extraction Techniques. *Environ. Sci. Technol.* **1994**, *28* (9), 1698–1705. DOI: 10.1021/es00058a023.

(10) Lueder, U.; Maisch, M.; Laufer, K.; Jorgensen, B. B.; Kappler, A.; Schmidt, C. Influence of Physical Perturbation on Fe(II) Supply in Coastal Marine Sediments. *Environmental Science & Technology* **2020**, *54* (6), 3209–3218. DOI: 10.1021/acs.est.9b06278.

(11) Martin, M. Cutadapt removes adapter sequences from high-throughput sequencing reads. *EMBnet j.* **2011**, *17* (1), 10. DOI: 10.14806/ej.17.1.200.

(12) Callahan, B. J.; McMurdie, P. J.; Rosen, M. J.; Han, A. W.; Johnson, A. J. A.; Holmes, S. P. DADA2: High-resolution sample inference from Illumina amplicon data. *Nature Methods* **2016**, *13* (7), 581–583. DOI: 10.1038/nmeth.3869.

(13) Quast, C.; Pruesse, E.; Yilmaz, P.; Gerken, J.; Schweer, T.; Yarza, P.; Peplies, J.; Glöckner, F. O. The SILVA ribosomal RNA gene database project: improved data processing and web-based tools. *Nucleic Acids Research* **2013**, *41* (Database issue), D590-6. DOI: 10.1093/nar/gks1219.

(14) Bolyen, E.; Rideout, J. R.; Dillon, M. R.; Bokulich, N. A.; Abnet, C. C.; Al-Ghalith, G. A.; Alexander, H.; Alm, E. J.; Arumugam, M.; Asnicar, F.; Bai, Y.; Bisanz, J. E.; Bittinger, K.; Brejnrod, A.; Brislawn, C. J.; Brown, C. T.; Callahan, B. J.; Caraballo-Rodríguez, A. M.; Chase, J.; Cope, E. K.; Da Silva, R.; Diener, C.; Dorrestein, P. C.; Douglas, G. M.; Durall, D. M.; Duvallet, C.; Edwardson, C. F.; Ernst, M.; Estaki, M.; Fouquier, J.; Gauglitz, J. M.; Gibbons, S. M.; Gibson, D. L.; Gonzalez, A.; Gorlick, K.; Guo, J.; Hillmann, B.; Holmes, S.; Holste, H.; Huttenhower, C.; Huttley, G. A.; Janssen, S.; Jarmusch, A. K.; Jiang, L.; Kaehler, B. D.; Kang, K. B.; Keefe, C. R.; Keim, P.; Kelley, S. T.; Knights, D.; Koester, I.; Kosciolek, T.; Kreps, J.; Langille, M. G. I.; Lee, J.; Ley, R.; Liu, Y.-X.; Loftfield, E.; Lozupone, C.; Maher, M.; Marotz, C.; Martin, B. D.; McDonald, D.; McIver, L. J.; Melnik, A. V.; Metcalf, J. L.; Morgan, S. C.; Morton, J. T.; Naimey, A. T.; Navas-Molina, J. A.; Nothias, L. F.; Orchanian, S. B.; Pearson, T.; Peoples, S. L.; Petras, D.; Preuss, M. L.; Pruesse, E.; Rasmussen, L. B.; Rivers, A.; Robeson, M. S.; Rosenthal, P.; Segata, N.; Shaffer, M.; Shiffer, A.; Sinha, R.; Song, S. J.; Spear, J. R.; Swafford, A. D.; Thompson, L. R.; Torres, P. J.; Trinh, P.; Tripathi, A.; Turnbaugh, P. J.; Ul-Hasan, S.; van der Hooft, J. J. J.; Vargas, F.; Vázquez-Baeza, Y.; Vogtmann, E.; Hippel, M. von; Walters, W.; Wan, Y.; Wang, M.; Warren, J.; Weber, K. C.; Williamson, C. H. D.; Willis, A. D.; Xu, Z. Z.; Zaneveld, J. R.; Zhang, Y.; Zhu, Q.; Knight, R.; Caporaso, J. G. Reproducible, interactive, scalable and extensible microbiome data science using QIIME 2. *Nature Biotechnology* **2019**, *37* (8), 852–857. DOI: 10.1038/s41587-019-0209-9.

(15) Caporaso, J. G.; Lauber, C. L.; Walters, W. A.; Berg-Lyons, D.; Lozupone, C. A.; Turnbaugh, P. J.; Fierer, N.; Knight, R. Global patterns of 16S rRNA diversity at a depth of millions of sequences per sample. *PNAS* **2011**, *108 Suppl 1* (Supplement 1), 4516–4522. DOI: 10.1073/pnas.1000080107.

(16) Stults, J. R.; Snoeyenbos-West, O.; Methe, B.; Lovley, D. R.; Chandler, D. P. Application of the 5' fluorogenic exonuclease assay (TaqMan) for quantitative ribosomal DNA and rRNA analysis in sediments. *Applied and Environmental Microbiology* **2001**, *67* (6), 2781–2789. DOI: 10.1128/AEM.67.6.2781-2789.2001.

(17) Bru, D.; Sarr, A.; Philippot, L. Relative abundances of proteobacterial membrane-bound and periplasmic nitrate reductases in selected environments. *Applied and Environmental Microbiology* **2007**, *73* (18), 5971–5974. DOI: 10.1128/AEM.00643-07.

(18) Henry, S.; Bru, D.; Stres, B.; Hallet, S.; Philippot, L. Quantitative detection of the nosZ gene, encoding nitrous oxide reductase, and comparison of the abundances of

16S rRNA, narG, nirK, and nosZ genes in soils. *Applied and Environmental Microbiology* **2006**, *72* (8), 5181–5189. DOI: 10.1128/AEM.00231-06.
(19) Sultana, M.; Vogler, S.; Zargar, K.; Schmidt, A.-C.; Saltikov, C.; Seifert, J.; Schlömann, M. New clusters of arsenite oxidase and unusual bacterial groups in enrichments from arsenic-contaminated soil. *Archives of Microbiology* **2012**, *194* (7), 623–635. DOI: 10.1007/s00203-011-0777-7.
(20) Song, B.; Chyun, E.; Jaffé, P. R.; Ward, B. B. Molecular methods to detect and monitor dissimilatory arsenate-respiring bacteria (DARB) in sediments. *FEMS Microbiology Ecology* **2009**, *68* (1), 108–117. DOI: 10.1111/j.1574-6941.2009.00657.x.
